# Resistance to 6-Methylpurine is Conferred by Defective Adenine Phosphoribosyltransferase in *Tetrahymena*

**DOI:** 10.3390/genes9040179

**Published:** 2018-03-23

**Authors:** Takahiko Akematsu, Andrew Findlay, Yasuhiro Fukuda, Ronald E. Pearlman, Josef Loidl, Eduardo Orias, Eileen P. Hamilton

**Affiliations:** 1 Department of Chromosome Biology, University of Vienna, 1030 Vienna, Austria; josef.loidl@univie.ac.at; 2 Department of Molecular, Cellular and Developmental Biology, University of California at Santa Barbara, Santa Barbara, CA 93106, USA; arfindlay@wustl.edu (A.F.); eduardo.orias@lifesci.ucsb.edu (E.O.); eileen.hamilton@lifesci.ucsb.edu (E.P.H.); 3 Department of Biodiversity Science, Division of Biological Resource Science, Graduate School of Agricultural Science, Tohoku University, Oosaki 989-6711, Japan; yasuhiro.fukuda.b7@tohoku.ac.jp; 4 Department of Biology, York University, Toronto, ON M3J 1P3, Canada; ronp@yorku.ca

**Keywords:** 6-methylpurine, adenine phosphoribosyltransferase, mutation, selection marker, *Tetrahymena thermophila*

## Abstract

6-methylpurine (6mp) is a toxic analog of adenine that inhibits RNA and protein synthesis and interferes with adenine salvage mediated by adenine phosphoribosyltransferase (APRTase). Mutants of the ciliated protist *Tetrahymena thermophila* that are resistant to 6mp were isolated in 1974, but the mechanism of resistance has remained unknown. To investigate 6mp resistance in *T. thermophila*, we created 6mp-resistant strains and identified a mutation in the APRTase genomic locus (*APRT1*) that is responsible for 6mp resistance. While overexpression of the mutated *APRT1* allele in 6mp-sensitive cells did not confer resistance to 6mp, reduced wild-type *APRT1* expression resulted in a significant decrease in sensitivity to 6mp. Knocking out or reducing the expression of *APRT1* by RNA interference (RNAi) did not affect robust cell growth, which indicates that adenine salvage is redundant or that de novo synthesis pathways provide sufficient adenosine monophosphate for viability. We also explored whether 6mp resistance could be used as a novel inducible selection marker by generating 6mp- and paromomycin-resistant double mutants. While 6mp- and paromomycin-resistant double mutants did express fluorescent proteins in an RNAi-based system, the system requires optimization before 6mp resistance can be used as an effective inducible selection marker.

## 1. Introduction

The ciliated protist *Tetrahymena thermophila* is a powerful model system that can be used to investigate fundamental cell and molecular structures and processes [1] such as self-splicing introns [2], small RNA-mediated genome rearrangement [3], telomeres and telomerase [4], meiosis in the absence of a synaptonemal complex [5], and selective nuclear autophagy [6]. A greater variety of selection markers for studying gene function and gene product localization would further enhance the utility of *T. thermophila*. However, at present selection markers are limited to drug resistance markers against paromomycin/neomycin [7,8], blasticidin [9,10], puromycin [11], and cycloheximide [12,13].

**Figure 1:**
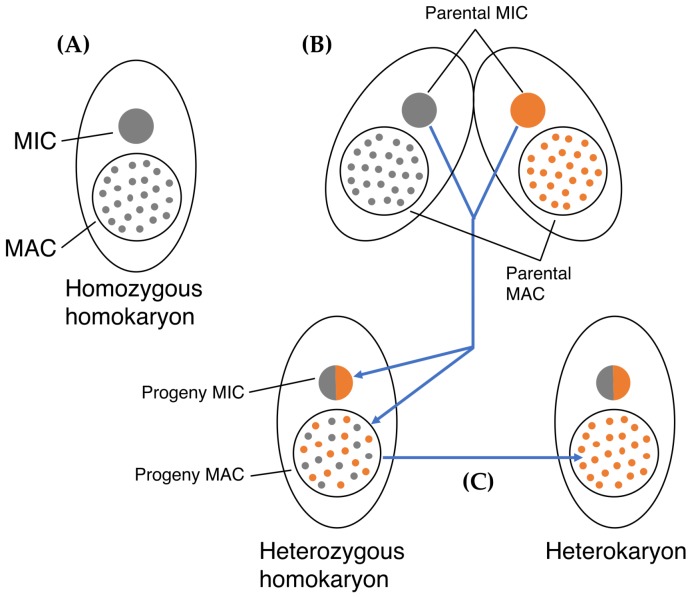
Cartoon illustrating nuclear dualism and inheritance in *Tetrahymena thermophila*. (**A**) Cell with a diploid micronucleus (MIC) and polyploid macronucleus (MAC). A cell is called a homokaryon if MIC and MAC carry the same alleles. (**B**) During sexual reproduction, two cells of different mating type pair (fuse at the anterior end). The MIC in each mate undergoes meiosis; in each mate three meiotic products are destroyed, while the remaining product divides to form two pronuclei. Mates exchange pronuclei, which then fuse resulting in a diploid zygotic nucleus. This nucleus divides giving rise to the new MICs and MACs; at the same time the parental MAC are destroyed. (**C**) Random assortment of MAC chromosomes over repeated amitotic divisions can result in homozygosity of the MAC whereas the diploid MIC, which divides by mitosis, remains heterozygous. A cell with a MIC and a MAC that have different genetic compositions is called a hetetokaryon.

As other ciliates, *T. thermophila* maintains differentiated germline and somatic nuclear genomes in a single cytoplasm [14]. The small, essentially transcriptionally silent diploid micronucleus (MIC) contains the germline genome, while the large, transcriptionally active polyploid macronucleus (MAC) houses the somatic genome. The phenotype of a cell, therefore, depends on the genetic constitution of the MAC, whereas only the genetic makeup of the MIC is inherited to progeny MICs and MACs during sexual reproduction (illustrated in Figure 1). Unlike the MIC, in which the chromosomes are mitotically distributed into the daughter nuclei, the MAC divides amitotically. Thus, MAC chromosomes are randomly segregated at every cell fission [15,16].

**Figure 2:**
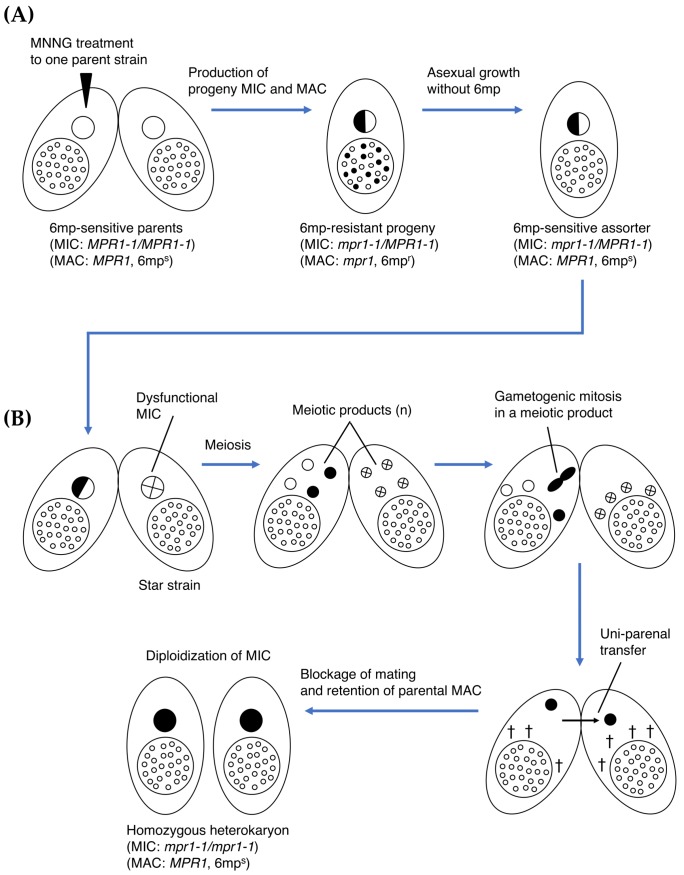
Cartoon illustrating processes leading to functional homozygous heterokaryons in *T. thermophila*. (**A**) *N*-methyl-*N*′-nitro-*N*-nitrosoguanidine (MNNG) mutagenesis was used to induce mutation(s) in the parental MIC (*MPR1-1/MPR1-1*) of one parent strain to create a 6mp-resistant (*mpr1*, 6mp^r^) heterozygous (*mpr1-1/MPR1-1*) progeny cell (Byrne et al. [19]). Numerous rounds of single-cell isolation, followed by culturing in the absence of 6mp established 6mp-sensitive (*MPR1*, 6mp^s^) assorters, which retain the heterozygous MIC (*mpr1-1/MPR1-1*). (**B**) The heterozygous heterokaryon was mated to a star strain with a dysfunctional MIC to obtain functional homozygous heterokaryons (*mpr1-1/mpr1-1*, [*MPR1*, 6mp^s^]) (Bruns et al. [20]). Non-star side cells are widely used in laboratories as pedigreed wild-type strains. Black and white nuclei indicate 6mp-resistant and -sensitive genotypes, respectively. † denotes unselected haploid nuclei MICs (pronuclei) destined to degrade.

Wild-type *T. thermophila* strains are sensitive to 6-methylpurine (6mp), a toxic adenine analog that inhibits enzymes that bind to adenine [17,18]. Byrne et al. [19] used *N*-methyl-*N*′-nitro-*N*-nitrosoguanidine (MNNG) mutagenesis to create a 6mp-resistant (6mp^r^) progeny cell by introducing mutation(s) in the parental MIC (illustrated in Figure 2A). Backcrosses to a 6mp-sensitive (*MPR1-1/MPR1-1*, 6mp^s^) wild-type cells indicated that the MIC genotype was heterozygous (*mpr1-1*/*MPR1-1*), which demonstrates that heterozygosity in the MAC is sufficient to attenuate the toxic activity of 6mp. Random assortment of alleles in the MAC enabled the establishment of 6mp^s^ assorters, which are phenotypically sensitive to the drug but retain the heterozygous MIC (*mpr1-1*/*MPR1-1*). Numerous rounds of single-cell isolation, followed by culturing in the absence of 6mp, enabled assortment (Figure 2A). Bruns et al. [20] mated these heterozygous heterokaryons (*mpr1-1*/*MPR1-1*, 6mp^s^) to a star (*) strain (i.e., a specialized *T. thermophila* strain with a defective MIC [21]) and obtained functional homozygous heterokaryons (*mpr1-1/mpr1-1*, 6mp^s^) through the first round of mating in genomic exclusion [1] (Figure 2B). These engineered strains are widely used in laboratories to simplify genetic analysis, as they allow for direct selection of sexual progeny cells. However, the nature of the mutation(s) induced by MNNG has not yet been characterized.

Two 6mp^r^ mutant strains have been described in the red bread mold *Neurospora crassa* [22]. Although the nature of 6mp resistance in these strains is unknown, Pendyala et al. [22] detected defective activity of adenine phosphoribosyltransferase (APRTase), which catalyzes phosphoribosyl transfer from phosphoribosyl pyrophosphate (PRPP) to adenine to produce adenosine monophosphate (AMP) [23]. Zhang et al. [24] found 6mp^r^ strains in natural isolates of the hyperthermophilic archaeon *Sulfolobus islandicus*. These strains carry mutations in the APRTase gene *APRT* [24]. Moreover, knocking out the gene in a 6mp^s^ strain resulted in a 6mp^r^ phenotype [24], strongly suggesting that impaired APRTase activity prevents the conversion of 6mp to the nucleotide form.

In the present study, we induced mutation(s) in the *APRT1* locus of *T. thermophila* 6mp^r^ strains. We also overexpressed this gene in a 6mp^s^ strain, and inactivated it by knockout and knockdown. Our results provide the first genetic evidence in a eukaryote of a relationship between APRTase and the 6mp^r^ phenotype. Finally, we investigated the utility of *APRT1* as a new selectable drug-resistance marker.

## 2. Materials and Methods

### 2.1. Strains, Culture Conditions, and Cell Counting

*T. thermophila* strains CU369 (*mpr1-1/mpr1-1* [*MPR1*; 6mp^s^, mating type IV]), CU428.2 (*mpr1-1/mpr1-1* [*MPR1*; 6mp^s^, mating type VII]), B2086.2 (*MPR1-1/MPR1-1* [*MPR1*; 6mp^s^, mating type II]), and A*V (a star strain that lacks a genetically functional micronucleus, mating type V) were obtained from the Tetrahymena Stock Center at Cornell University (USA) (https://tetrahymena.vet.cornell.edu/). A strain (*MPR1-1/MPR1-1* [*MPR1*; 6mp^s^]) that expresses histone H3 with a C-terminal mCherry fluorescent protein tag (HHT-mCherry) was a gift from Dr. Kensuke Kataoka (National Institute for Basic Biology, Okazaki, Japan). Cells were grown at 30 °C in super proteose peptone (SPP) medium containing 1% proteose peptone (Becton Dickinson, Sparks, MD, USA), 0.1% yeast extract (Becton Dickinson), 0.2% glucose (Sigma-Aldrich, St. Louis, MO, USA), and 0.003% EDTA-Fe (Sigma-Aldrich). 6mp (Sigma-Aldrich) dissolved in distilled water at 15 mg/mL was used as a stock solution. For growth curves, approximately 5 × 10^4^ cells were inoculated in 1 mL of SPP containing 15 µg/mL 6mp in 1.5 mL microcentrifuge tubes (Eppendorf, Hamburg, Germany). Cell viability was determined with a hemocytometer (Sigma-Aldrich) under a stereo microscope. Since dead cells accumulated at the bottom of the growth tube, we collected a 10 µL aliquot with swimming cells from the surface of the growth medium. For counting, cells were immobilized by adding 0.5 µL of 37% formaldehyde (Sigma-Aldrich).

### 2.2. Genomic Exclusion

To obtain homozygous homokaryons (*mpr1-1/mpr1-1* [*mpr1*; 6mp^r^]), we crossed CU369 (mating-type IV) and CU428.2 (mating-type VII) cells with A*V (mating-type V) cells. To make them competent for mating, cells at mid-log phase (approximately 10^6^/mL) were washed with 10 mM Tris-HCl (pH 7.4), resuspended in 10 mM Tris-HCl (pH 7.4), and starved at 30 °C for approximately 18 h. To induce mating, we mixed together equal numbers of cells of each mating type and incubated at 30 °C. During the mating, one directional transfer of the migratory haploid gametic pronucleus occurred from the wild-type parent to the star parent, followed by the blockage of mating without the production of progeny nuclei. After refeeding the pairs with SPP, the haploid pronuclei underwent self-diploidization, resulting in both parental cells having identical, functional, diploid whole genome-homozygous MICs [1]. The parental cells retained their old MAC and hence their mating types. We isolated cells of these two mating types and induced a second round of mating between them to produce progeny. We selected progeny cells with MACs homozygous for 6mp^r^ by cultivating in SPP containing 15 μg/mL 6mp.

### 2.3. Cloning and Sequencing of APRT1

*APRT1* open reading frames (ORFs) were amplified from genomic DNA of the homozygous homokaryons (*mpr1-1/mpr1-1* [*mpr1*; 6mp^r^]), as well as from wild-type B2086.2 cells, with PrimeSTAR Max DNA polymerase (Takara, Kusatsu, Japan) and the following primers: forward, GAGCTGAATGAATGAATGAATGAAT and reverse, CGTTTATTTATGACCTTTGACATCC. Amplified polymerase chain reaction (PCR) products were integrated into the EcoRV site of the pBluescript SK(+) vector with T4 DNA ligase (New England BioLabs, Ipswich, MA, USA). Cloned PCR products in the plasmids were sequenced at LGC Genomics (Berlin, Germany) with the M13R primer, CAGGAAACAGCTATGAC. High-efficiency DH5 alpha-competent *Escherichia coli* cells (New England BioLabs) were used to amplify all plasmids created in this study.

### 2.4. Construction of C-Terminal Epitope-Tagged Vectors

Plasmid pFZZ-NEO4 [25] contains FZZ (three tandem repeats of the FLAG epitope, a TEV protease-sensitive site, and a protein A epitope) and a *NEO4* drug-resistant marker [8]. To create double-mutant strains (described in Section 3.4), we replaced the *NEO4* cassette with a puromycin resistance marker (*PAC*) [11]. The *NEO4* cassette was removed from the plasmid by digestion with SalI and XmaI (New England BioLabs). pTOP2Gi-PAC [26] carries *PAC* under the control of the *MTT2* copper-inducible promoter [27] between SalI and XmaI sites. The *MTT2-PAC* cassette was excised from pTOP2Gi-PAC and integrated into the SalI–ApaI sites of pFZZ-NEO4 with T4 DNA ligase (New England BioLabs). The resulting plasmid (pFZZ-PAC) was used as a backbone plasmid for further constructs. Approximately 1 kb of the ORF (5′) and untranslated region (UTR) (3′) of the *APRT1* genomic locus were amplified from the genomic DNA of the homozygous homokaryons *mpr1-1/mpr1-1* (*mpr1*; 6mp^r^) with PrimeSTAR Max DNA polymerase (TaKaRa) and the following primers: ORF 5′ forward, ctaaagggaacaaaagctggagctcGAGCTGAATGAATGAATGAATGAAT; ORF 5′ reverse, accatcatgatctttgtaatcggatccTTCAATATGAAGATTAATAACCT; UTR 3′ forward, aatgcagaagcttgtcgacctcgagGAATGAACAAGTTAGTTTGTTTTTG; and UTR 3′ reverse, ctcactatagggcgaattgggtaccCGTTTATTTATGACCTTTGACATCC. The nucleotides in lowercase correspond to consensus sequences with the DNA ends of the backbone. The 5′ ORF sequence did not contain a stop codon. The amplified PCR products were sequentially integrated into the SacI–BamHI sites and XhoI–KpnI sites, respectively, of pFZZ-PAC with the NEBuilder HF DNA Assembly Kit (New England BioLabs). The resulting plasmid (pD127-FZZ-PAC, Figure S1) was linearized with SacI and KpnI (New England BioLabs) before biolistic transformation into *T. thermophila* [28]. The control plasmid, which carried wild-type *APRT1* instead of *APRT1* from 6mp^r^ cells, was also prepared. The PAC cassette was activated by adding 630 µM CuSO_4_ to the transformed cells. Once puromycin-resistant transformants were obtained, the concentration of CuSO_4_ was reduced to 20 µM for the efficient induction of phenotypic assortment. Western blotting for tagged proteins was performed as described previously [26]. Total protein (10 µg) extracted from growing cells in SPP medium was loaded into each lane of a 4–15% precast polyacrylamide gel (Bio-Rad, Hercules, CA, USA), separated by sodium dodecyl sulfate-polyacrylamide gel electrophoresis (SDS-PAGE), and transferred onto polyvinylidene fluoride membrane (Bio-Rad). Anti-FLAG antibody (1:5000; Sigma-Aldrich) or anti-tubulin-alpha antibody (1:10,000; NeoMarkers, Fremont, CA, USA) were used as primary antibodies. 

### 2.5. Construction of Macronucleus (MAC) APRT1 Disruption Vector

Approximately 1 kb of sequences upstream (5′) and downstream (3′) of the macronuclear *APRT1* genomic locus were amplified from B2086.2 genomic DNA with PrimeSTAR Max DNA polymerase (Takara) and the following primers: 5′ forward, ctaaagggaacaaaagctggagctcGAGCTGAATGAATGAATGAATGAAT; 5′ reverse, aagatatcaagtcgacgcggccgccAATTTTATTACCCCACAAATCAATC; 3′ forward, atgcagcccgggggatcagctcgagGGATCTTAAGGGCGAAGAAAGATTA; and 3′ reverse, ctcactatagggcgaattgggtaccCGTTTATTTATGACCTTTGACATCC. Amplified PCR products were sequentially integrated into SacI–NotI sites and XhoI–KpnI sites of pNEO5 (a gift from Dr. Kazufumi Mochizuki, Institute of Human Genetics, Montpellier, France) with the NEBuilder HF DNA Assembly Kit (New England BioLabs). The resulting plasmid (pΔAPRT1-NEO5, Figure S2) was linearized with SacI and KpnI (New England BioLabs) before biolistic transformation into *T. thermophila*.

### 2.6. APRT1 RNA Interference (RNAi) Vector Construction and Gene Knockdown

T4 DNA ligase (New England BioLabs) was used to integrate double-stranded DNA (1146 bp) that comprised two junk sequences separated by a 103-bp linker sequence (synthesized at Integrated DNA Technologies, Coralville, IA, USA) into the PCR product amplified from pMTT1-D127N-NEO5 (Figure S3) with PrimeSTAR Max DNA polymerase (Takara) and the following primers: forward, AAATAATAATACTAAACTTAAACAT and reverse, ACTAGTTGAGCGAACTGAATCGGTC, which had been 5′ phosphorylated by T4 polynucleotide kinase (New England BioLabs). Figure S4 shows the entire sequence of the resulting plasmid (pAkRNAi-NEO5). Target sequences used in hairpin constructs (537 bp of the *APRT1* ORF) were amplified from B2086.2 genomic DNA with PrimeSTAR Max DNA polymerase (Takara) and the following primers: 5′ forward, taaacttaaacatcccgggggatccGGATTACTAAAATTAAGAAATAGGT; 5′ reverse, ttgcatatccgttacttacggatccCATTCAATATGAAGATTAATAACCT; 3′ forward, taaaagaagaattcaaaggctgcagCATTCAATATGAAGATTAATAACCT; and 3′ reverse, gctgaccgattcagttcgcctgcagGGATTACTAAAATTAAGAAATAGGT. Amplified forward and reverse target fragments were cloned into the BamHI–BamHI and PstI–PstI I sites, respectively, of pAkRNAi-NEO5 with the NEBuilder HF DNA Assembly Kit (New England BioLabs) to create the hairpin cassette. The resulting plasmid (pAPRT1i-NEO5, Figure S5) was linearized with SacI and KpnI (New England BioLabs) before biolistic transformation into *T. thermophila*. RNA interference (RNAi) was induced in cells carrying the hairpin construct by adding 1 µg/mL CdCl_2_ (Sigma-Aldrich) to promote double stranded RNA (dsRNA) expression from the *MTT1* cadmium-inducible promoter [29].

### 2.7. Southern Blotting

Genomic DNA samples (5 µg) digested with BamHI and XhoI (New England BioLabs) were electrophoresed through an 0.8% agarose gel and then transferred by capillary action onto a Hybond-nylon membrane (GE Healthcare, Little Chalfont, UK) with 20× SSPE (3 M NaCl, 20 mM EDTA, 154.8 mM Na_2_HPO_4_, and 45.2 mM H_6_NaO_6_P; pH 7.4). For the probe, a 579-bp PCR product was amplified from B2086.2 wild-type genomic DNA with the following primers: forward, GAGCTGAATGAATGAATGAATGAAT and reverse, AATTTTATTACCCCACAAATCAATC. The probe was radioactively labeled by random priming with ^32^P-dATP (Hartmann Analytic, Braunschweig, Germany) and hybridized to the DNA on the membrane. The signal was detected with an imaging plate (FUJIFILM Corporation, Tokyo, Japan) and scanned with a Typhoon 9200 image analyzer (GE Healthcare). Band intensity (a rough estimation of the degree of MAC assortment) was measured using ImageJ software.

### 2.8. Construction of EGFP-Tagged ATG8.2 Expression Vectors Based on pD127-FZZ-PAC

The *ATG8.2* ORF was amplified from B2086.2 genomic DNA with PrimeSTAR Max DNA polymerase (Takara) and the following primers: forward, ggatgaattatataagggatccATGGACGCTCAAAATTATAAACCC and reverse, cgattcagttcgctcaactagtAATTGATCCAAATACTTCTTGATCAGAG. The amplified PCR product was integrated into the BamHI–SpeI sites of pBNMB1-EGFP with the NEBuilder HF DNA Assembly Kit (New England BioLabs). The resulting plasmid (pEGFP-ATG8.2-NEO5) contained a polyadenylation (polyA) signal followed by the *MTT1* promoter, the *EGFP* cassette, the *ATG8.2* ORF, and the 3′ UTR of *BTU1*. These consecutive components were amplified as a single fragment with the following primers: forward, tattaatcttcatattgaaggatccTGAGATCCTTAAATTAAAAATTCAATAT and reverse, aacttgttcattcctcgaggtcgacAAGATGTGGCTATTGATGGGCATAA. The amplified fragment was integrated into the BamHI–SalI sites of pD127-FZZ-PAC with the NEBuilder HF DNA Assembly Kit (New England BioLabs). The resulting plasmid (pD127-EGFP-ATG8.2, Figure S6) was linearized with SacI and KpnI (New England BioLabs) before biolistic transformation into *T. thermophila*.

### 2.9. Construction of EGFP-Tagged ATG8.2 Expression Vectors Based on pΔAPRT1-NEO5

A DNA fragment containing the *MTT1* promoter followed by the *EGFP* cassette, the *ATG8.2* ORF, and the 3′ UTR of the *BTU1* gene was amplified from pEGFP-ATG8.2-NEO5 with PrimeSTAR Max DNA polymerase (Takara) and the following primers: forward, ataaacaaaatccagatcccccgggGGATCAGACAATTTATTTCTAAAAA and reverse, aacttgttcattcctcgaggtcgacAAGATGTGGCTATTGATGGGCATAA. The amplified fragment was integrated into the XmaI–SalI sites of pΔAPRT1-NEO5 with the NEBuilder HF DNA Assembly Kit (New England BioLabs). The resulting plasmid (pEGFP-ATG8.2 APRT1 locus, Figure S7) was linearized with SacI and KpnI (New England BioLabs) before biolistic transformation into *T. thermophila*.

### 2.10. Construction of EGFP-Tagged TOP2S Expression Vectors Based on pAPRT1i-NEO5

A DNA fragment containing the *NEO5* cassette followed by the *MTT1* promoter, the hairpin RNAi region, and the 3′ portion of the *BTU1* genomic locus was excised from pAPRT1i-NEO5 with SalI and ApaI (New England BioLabs). It was integrated into the SalI–ApaI sites of pTOP2S-EGFP-NEO4 [26] with T4 DNA ligase (New England BioLabs). The resulting plasmid (pTOP2S-EGFP-NEO5-APRT1i, Figure S8) was linearized with SacI and KpnI (New England BioLabs) before biolistic transformation into *T. thermophila*. The double mutant carried both TOP2S-EGFP and HHT-mCherry, as confirmed by PCR with the diagnostic primers TOPS-EGFP forward, ACGCTAAGGAGCAGACCTCG; TOPS-EGFP reverse, ACTAGTCTTATATAATTCATCCATA; HHT-mCherry forward, ATGGCTAGAACTAAATAAACTGCTA; and HHT-mCherry reverse, GAATATCCCATGCGAAAGGTAAAGG. 

## 3. Results and Discussion

### 3.1. Mutation in APRT1 of 6mp^r^ Strains

APRTase plays a critical role in adenine salvage in most living organisms [23]. The phosphoribosyltransferase (PRTase) protein family catalyzes Mg^2+^-dependent replacement of the 1-pyroposphate group from PRPP with a purine base to form the corresponding purine nucleoside 5′-monophosphate. In many organisms, purine nucleotides, including AMP, can be generated via a de novo synthetic pathway; in addition, several purine salvage pathways incorporate preformed purine bases and nucleosides into the purine nucleotide pool.

**Figure 3:**
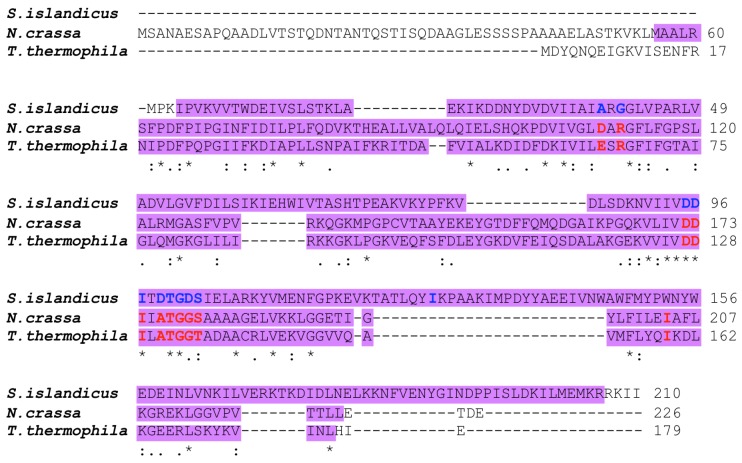
Multiple-sequence alignment of the entire amino acid sequences of *Sulfolobus islandicus*, *Neurospora crassa*, and *T. thermophila* adenine phosphoribosyltransferases (APRTases). Purple boxes indicate the phosphoribosyltransferase (PRTase)-type I domain. Amino acids in blue and red correspond to putative active sites important for prokaryotic and eukaryotic APRTase functions, respectively. Asterisks indicate identical amino acids. Colons and semicolons indicate amino acid similarity.

APRTase has been implicated in resistance to 6mp in *N. crassa* [22] and the archaeon *S. islandicus* [24]. A BLASTP search of the *T. thermophila* genome database (http://www.ciliate.org/) revealed that TTHERM_00011180 (*APRT1*) alone encodes a protein similar to the eukaryotic *N. crassa* APRTase (GenBank: EAA34491.3). This potential APRTase contains a PRTase-type I domain, a characteristic of PRPP-binding proteins, and putative active sites important for enzyme function (Figure 3). Similarly, *S. islandicus* APRTase (ACR40792.1) shares the PRTase-type I domain, as well as some of the putative active sites, with eukaryotic APRTases (Figure 3). However, the entire sequence of *S. islandicus* APRTase is similar to prokaryotic PRTases with specificities for hypoxanthine, guanine, xanthine, and/or adenine, which are not conserved in *T. thermophila* or *N. crassa* (http://fungi.ensembl.org/Neurospora_crassa/Info/Index), implying that the eukaryotic and prokaryotic APRTases work as isozymes. Indeed, a gene (*TK0664*) encoding hypoxanthine–guanine PRTase has been used as a 6mp counterselective marker in the archaeon *Thermococcus barophilus* [30].

**Figure 4:**
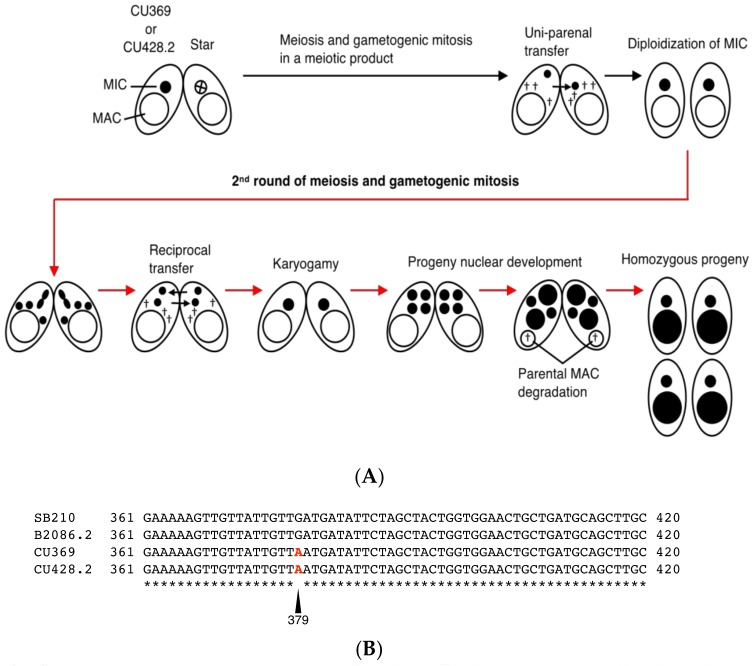

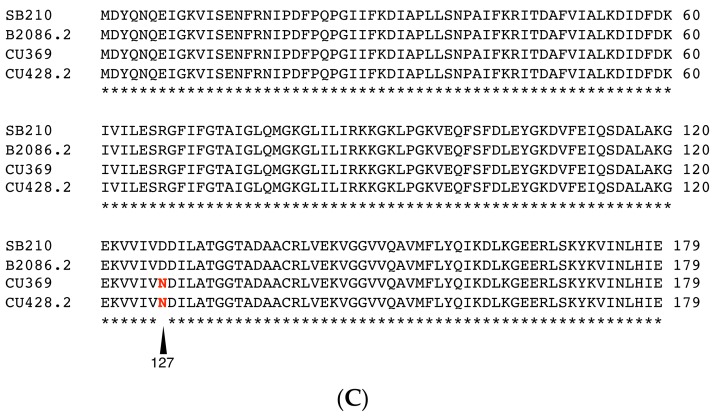
Identification of a mutation in *APRT1*. (**A**) Diagram illustrating the process of genomic exclusion to generate homozygous homokaryons. Star strains possess a dysfunctional MIC, represented by a cross. Black and red arrows correspond to the first and second rounds of mating, respectively. Black and white nuclei indicate 6mp-resistant and -sensitive genotypes, respectively. † denotes nuclei destined to degrade, such as unselected haploid MICs and old parental MACs. (**B**) Multiple-sequence alignment of the sequenced *APRT1* genes, including B2086.2 and homozygous homokaryons derived from CU369 and CU428.2. SB210 corresponds to the sequence obtained from the *Tetrahymena* genome database. Asterisks indicate identical nucleotides. Nucleotides in red at position 379 denote a common mutation found in this study. (**C**) Translation of the sequenced *APRT1* genes. Asterisks indicate identical amino acids. The amino acid change caused by the mutation at position 127 is indicated in red.

*T. thermophila* strains CU369 and CU428.2 are both functional homozygous heterokaryons (*mpr1-1/mpr1-1* [6mp^s^]) derived from the natural isolate B strain [31]; both carry the allele for 6mp-resistance that was induced by MNNG-treatment of mating cells [19]. To search for mutation(s) at the *APRT1* genomic locus, we created homozygous homokaryons (*mpr1-1/mpr1-1* [6mp^r^]) by mating CU369 and CU428.2 to star strain A*V for two rounds of genomic exclusion, an aberrant form of mating that creates whole-genome homozygotes (Figure 4A). We cloned the *APRT1* ORF and found a mutation at position 379, a change from guanine to adenine (Figure 4B). This mutation causes an amino acid change at position 127 from aspartic acid to asparagine (Figure 4C), which removes a conserved putative active site found in both the prokaryotic and eukaryotic enzymes (Figure 3). This result suggests that the mutation in *APRT1* is responsible for 6mp resistance.

### 3.2. Expression of D127N from APRT1 Genomic Locus Confers 6mp Resistance

**Figure 5:**
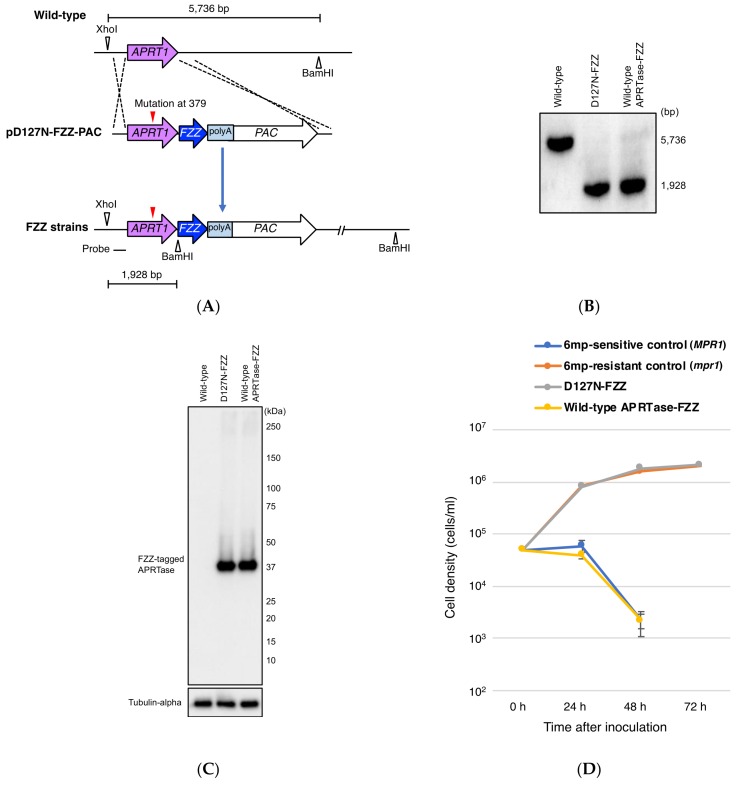
Effect on cell sensitivity to 6mp of replacing wild-type *APRT1* with the mutant gene. (**A**) A schematic showing the *APRT1* genomic locus (upper), the plasmid vector pD127N-FZZ-PAC, with the mutant gene, FZZ tag containing polyA signal, and puromycin resistant cassette (*PAC*) (middle), and after homologous recombination (lower). Control plasmid carries wild-type *APRT1* instead of the mutated version. (**B**) Southern blot analysis of XhoI- and BamHI-digested genomic DNA from wild-type cells and FZZ-tagged APRTase-expressing transformants. The molecular weight of signals against the probe corresponds to the prediction in (**A**). (**C**) Western blot analysis of FZZ-tagged APRTases. FZZ tag and APRTase were 17 kDa and 20 kDa, respectively, resulting in a single 37 kDa band. Tubulin-alpha was the loading control. (**D**) Cell growth curves in the presence of 15 µg/mL 6mp. Points and attached bars correspond to mean measurements from three identical experiments and their standard deviations, respectively. Cells sensitive to 6mp all died by 72 h.

Resistance to 6mp in *N. crassa* [22] and the archaeon *S. islandicus* [24] has been suggested to result from inactivation of APRTase. Thus, we speculated as to whether mutation D127N affects *T. thermophila* APRTase activity. To test if D127N expression makes cells resistant to 6mp, we replaced wild-type MAC *APRT1* with mutated *APRT1* by integrating a C-terminal FZZ-tagged D127N gene-fusion product (from pD127-FZZ-PAC) into the macronuclear *APRT1* locus of B2086.2 cells (Figure 5A). As a control, a C-terminal FZZ-tagged wild-type *APRT1* gene-fusion product was also integrated into B2086.2 cells. Transformants were maintained in the presence of the selective agent, 2 mg/mL puromycin [11], for 2 months.

Southern blotting revealed that the original *APRT1* copies in the MAC had been completely replaced by the FZZ constructs (Figure 5B). Western blotting demonstrated that both wild-type and mutant transformants expressed FZZ-tagged proteins under the control of the endogenous *APRT1* promoter (Figure 5C).

To measure growth rate, we exposed both wild-type and mutant transformants to 15 µg/mL 6mp, which kills sensitive cells within 72 h [20,32]. B2086.2 (6mp^s^) and heterozygous progeny cells of a B2086.2 and CU428.2 mating (*mpr1-1*/*MPR1-1* [6mp^r^]) were used as 6mp-sensitive and -resistant control cells, respectively. Wild-type APRTase-FZZ-expressing cells were sensitive to 6mp, whereas D127N-FZZ-expressing cells grew similarly to 6mp^r^ control cells and had reached >10^6^ cells/mL by 72 h (Figure 3D). These results suggest that the D127N mutant protein has little to no enzyme activity, thus preventing the incorporation of a lethal dose of 6mp. Indeed, ectopic expression of D127N from a non-*APRT1* locus in B2086.2 cells did not confer 6mp resistance (Figure S3), suggesting that the resistance conferred by D127N is dependent upon wild-type APRTase dosage rather than upon the ratio of wild-type to mutant APRTase expression.

### 3.3. Partial Loss of MAC APRT1 Copies is Sufficient to Confer 6mp Resistance

**Figure 6:**
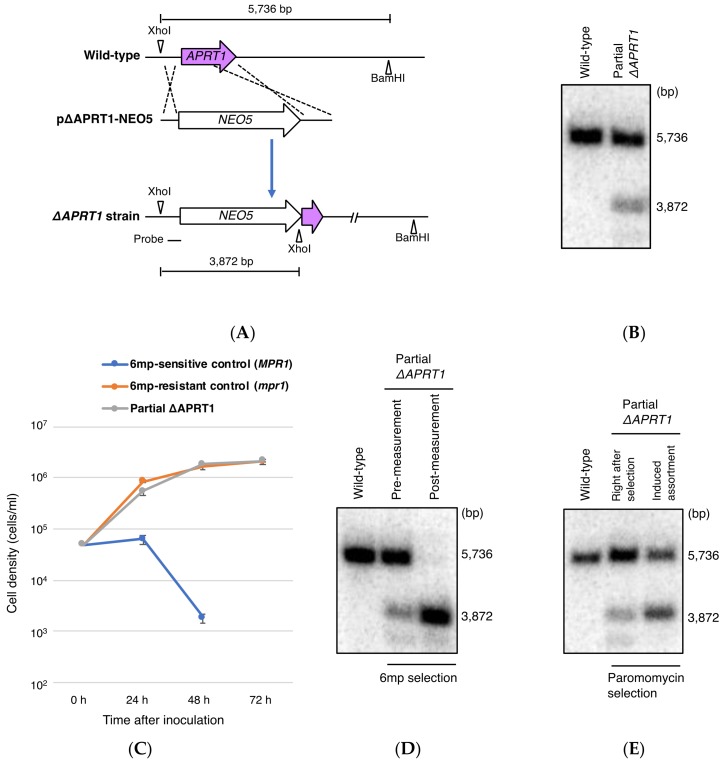
Effect of partial *APRT1* knockout on cell sensitivity to 6mp. (**A**) A schematic showing the *APRT1* genomic locus (upper), the plasmid vector pΔAPRT1-NEO5, with paromomycin resistance cassette (*NEO5*) (middle), and after homologous recombination (lower). (**B**) Southern blot analysis of XhoI- and BamHI-digested genomic DNA from wild-type cells and partial *APRT1* knockout cells. Molecular weight of signals against the probe corresponds to the prediction in (**A**). (**C**) Cell growth curves in the presence of 15 µg/mL 6mp. Points and attached bars correspond to mean measurements from three identical experiments and their standard deviations, respectively. Cells sensitive to 6mp all died by 72 h. (**D**) Southern blot analysis of XhoI- and BamHI-digested genomic DNA from partial *APRT1* knockout cells before and after measurement of cell growth rates in 6mp. (**E**) Southern blot analysis of XhoI-and BamHI-digested genomic DNA from partial *APRT1* knockout cells before and after the induction of phenotypic assortment with 5 mg/mL paromomycin for 2 months. Molecular weight of signals against the probe corresponds to that predicted in the schematic in (**A**).

Resistance to 6mp results from knockout of an *APRT* ortholog [24] in *S. islandicus* and complete replacement of wild-type *APRT1* with the D127N allele in *T. thermophila* (Figure 5D). However, ectopic overexpression of D127N did not cause resistance (Figure S3). These observations suggest that reduced numbers of wild-type *APRT1* copies, rather than D127N expression, are critical to 6mp resistance. To investigate this possibility, we integrated a somatic knockout construct (pΔAPRT1-NEO5) carrying the neomycin resistance marker *NEO5* into the MAC *APRT1* locus of wild-type B2086.2 cells (Figure 6A). After an initial 3-day drug selection with 100 μg/mL paromomycin (a neomycin derivative), the resulting transformant cells had not completed phenotypic assortment. Southern blotting revealed partial somatic *APRT1* knockout of approximately 30% of the copies (Figure 6B). 

When exposed to 15 μg/mL 6mp, the partial *APRT1* knockout cells grew similarly to 6mp^r^ control cells, reaching >10^6^ cells/mL at 72 h (Figure 6C). Southern blotting after 72 h showed complete loss of wild-type *APRT1* copies without adding paromomycin (Figure 6D). This is evidence of exceptionally quick phenotypic assortment compared to paromomycin selection: Even after 2 months of cultivation in 5 mg/mL paromomycin, assortment remained unfinished, with a ratio between the two *APRT1* alleles of approximately 1:1 (Figure 6E). This suggests that in *T. thermophila,* 6mp is a stronger negative selective agent than paromomycin. 

These results support the hypothesis that attenuated APRTase activity, due to the D127N mutation, is responsible for 6mp resistance. The initial partial loss of *APRT1* copies may confer limited resistance to 6mp and promote quick phenotypic assortment to allow survival under continuous selective pressure.

### 3.4. RNAi Against APRT1 Enables Survival in the Presence of 6mp

**Figure 7:**
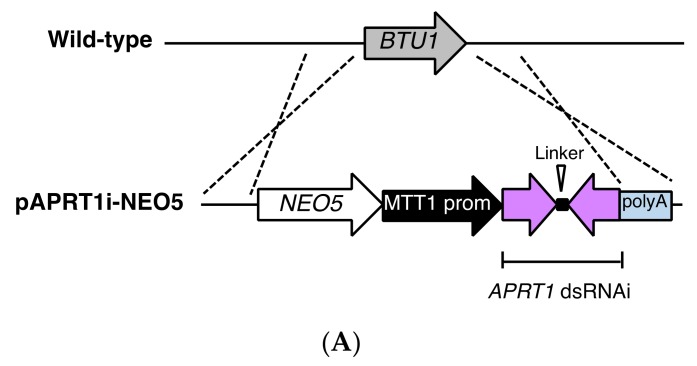

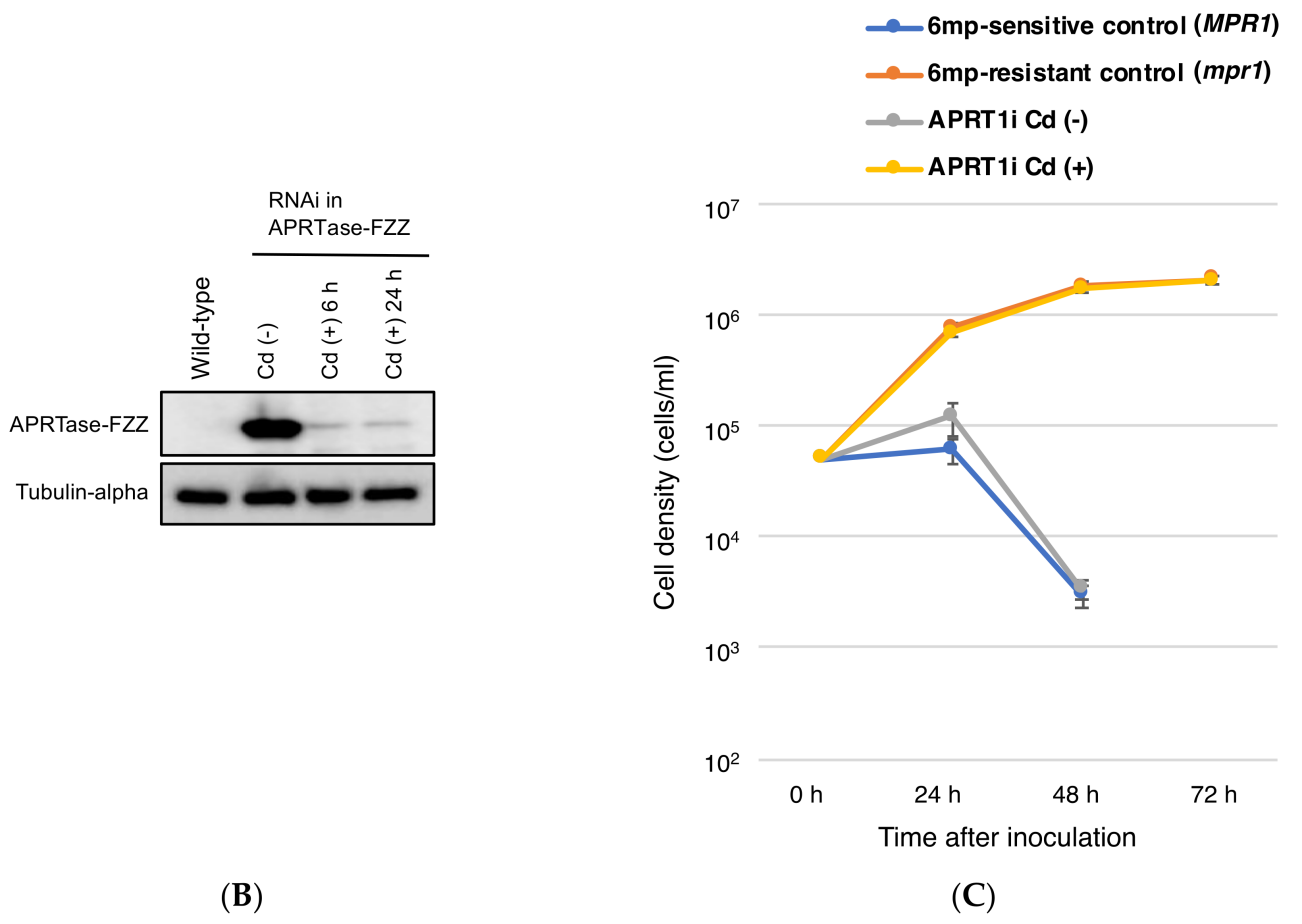
Effect of *APRT1* interference RNA (RNAi) on cell sensitivity to 6mp. (**A**) A schematic showing the *BTU1* genomic locus (upper), the plasmid vector pAPRT1i-NEO5, with paromomycin resistance cassette (*NEO5*), cadmium-inducible *MTT1* promoter, and hairpin RNAi cassette containing *BTU1*’s polyA signal (lower). (**B**) Western blot analysis of FZZ-tagged APRTase in RNAi non-induced and induced cells (exposed to 1 µg/mL CdCl_2_ in super proteose peptone (SPP) medium for 6 h or 24 h). Tubulin-alpha was the loading control. (**C**) Cell growth curves in 15 µg/mL 6mp. Points and attached bars correspond to the mean of three identical measurements and standard deviations. Cells sensitive to 6mp all died by 72 h.

To confirm that 6mp resistance in *T. thermophila* was due to reduced APRTase activity, we integrated a short hairpin RNAi construct (pAPRT1i-NEO5) controlled by the *MTT1* promoter into the MAC *BTU1* locus of B2086.2 cells (Figure 7A). The same construct was also introduced into the wild-type APRTase-FZZ-expressing strain (Figure 5C). Western blotting showed that APRTase-FZZ expression was largely abolished after cadmium induction of RNAi for 6 h (Figure 7B), which demonstrates that the RNAi construct was effective. The transformant cells became resistant to 15 μg/mL 6mp upon activation of RNA interference by 1 μg/mL CdCl_2_ (Figure 7C). This confirms that suppression of APRTase expression was responsible for the 6mp resistance.

In *S. islandicus*, addition of either AMP or guanosine monophosphate (GMP), both of which are non-essential in the wild-type strain, is required to maintain robust growth of the *APRT* knockout strain [24]. The utilization of GMP is probably due to the interconversion between GMP and AMP in purine metabolism, which has been observed in *Salmonella typhimurium* [33]. In the ciliate *T. thermophila*, we found that neither knockout nor knockdown of *APRT1* affected robust cell growth (Figure 6C and Figure 7C), which indicates that, unlike in prokaryotes and *Giardia lamblia*, in which adenine salvage provides the sole mechanism to produce AMP [34], APRTase is not required for cell growth. This independence from adenine salvage is similar to that seen in mammals, in which APRTase-dependent salvage is redundant with de novo adenosine synthesis (although it becomes more important during periods of vigorous cell growth, such as embryogenesis and tumor growth) [35]. Purine salvage appears to be redundant in *T. thermophila*, and sufficient AMP is most likely created through de novo synthesis.

### 3.5. Potential Utility of APRT1 as a New Selection Marker

**Figure 8:**
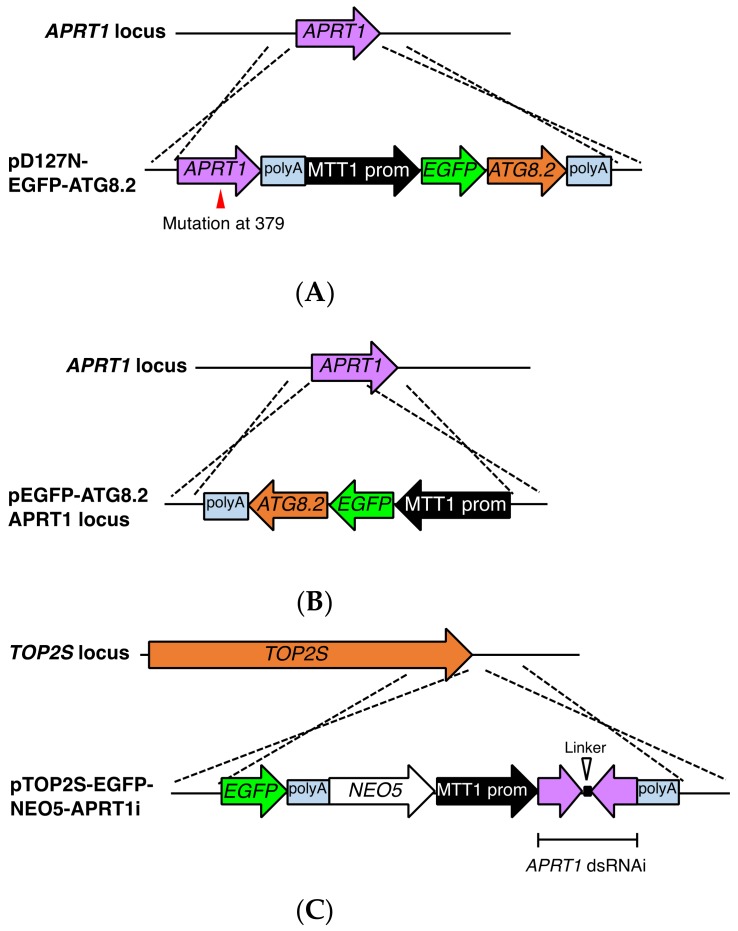
*APRT1* as a new selection marker. (**A**) A schematic diagram showing the *APRT1* genomic locus (upper) and the plasmid vector pD127N-EGFP-ATG8.2, with the mutated gene containing polyA signal and EGFP-tagged ATG8.2 overexpression cassette (lower). (**B**) A schematic diagram showing the *APRT1* genomic locus (upper) and the plasmid vector pEGFP-ATG8.2 APRT1 locus, with EGFP-tagged ATG8.2 overexpression cassette (lower). These constructs replace the functional *APRT1* gene with EGFP-ATG8.2 by homologous recombination. (**C**) A schematic diagram showing the *TOP2S* genomic locus (upper) and the plasmid vector pTOP2S-EGFP-NEO5-APRT1i, with EGFP tag containing polyA signal, paromomycin resistance cassette (*NEO5*), cadmium-inducible *MTT1* promoter, and hairpin RNAi cassette containing polyA signal (lower) with crossover sites for homologous recombination indicated. Selection is performed in the presence of cadmium. Once 6mp-resistant transformants are selected, the production of double stranded RNAs (dsRNAs) is inactivated by withdrawal of cadmium to minimize non-specific and off-target effects of the RNAi. The *NEO5* cassette maintains the construct by culturing the transformants with paromomycin, ultimately leading to homozygosity of the MAC. These plasmids were biolistically bombarded into an HHT-mCherry-expressing strain (*MPR1-1/MPR1-1*, 6mp^s^).

Our identification of *APRT1* as the gene responsible for 6mp resistance allowed us to add 6mp resistance to the set of available selectable drug resistance markers in *T. thermophila*. To evaluate whether selection of 6mp resistance could be used practically, we created three types of vectors: pD127N-EGFP-ATG8.2, pEGFP-ATG8.2 APRT1 locus, and pTOP2S-EGFP-NEO5-APRT1i (Figures S6–S8; described in Materials and Methods Section 2.8, Section 2.9 and Section 2.10). pD127N-EGFP-ATG8.2 and pEGFP-ATG8.2 APRT1 locus (*APRT1* locus-based systems) were designed to select for 6mp^r^ transformants by replacing the wild-type *ARPT1* copies in the MAC with either a non-functional (Figure 8A) or a deleted gene (Figure 8B), respectively. These constructs replace the functional *APRT1* gene with EGFP-ATG8.2, a fluorescently labeled protein that is targeted to autophagosomes in *T. thermophila* [36]. pTOP2S-EGFP-NEO5-APRT1i (RNAi-based system) was designed to suppress APRTase expression (resulting in 6mp resistance) upon induction of RNAi by cadmium treatment; once transformants are selected, transgenes can be maintained at the target locus through paromomycin resistance (Figure 8C). In strains that are already resistant to paromomycin, this approach could be used to select for secondary transformants. Withdrawal of cadmium after selection loading to inactivation of RNAi could minimize non-specific and off-target effects of the RNAi [37]. To test whether such double transformants could be generated, we biolistically bombarded each vector into a 6mp-sensitive (*MPR1-1*/*MPR1-1*) histone H3 (HHT)-mCherry-expressing strain in which the MAC carries a *NEO5* cassette following the *mCherry* cassette.

**Figure 9:**
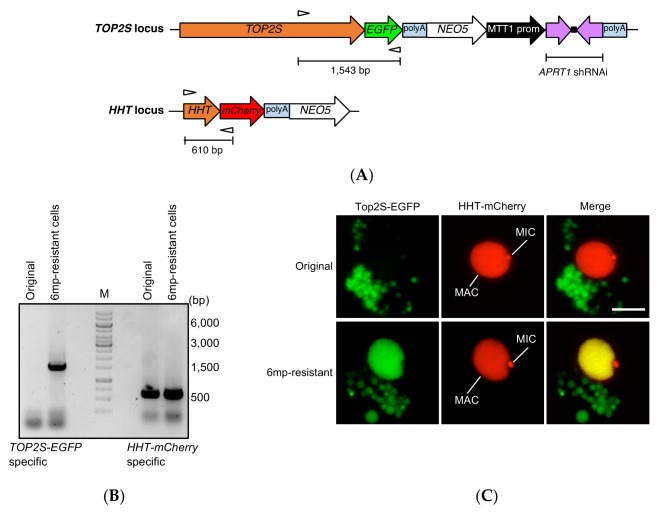
*APRT1* RNAi cassette acts as a 6mp selection marker. (**A**) Schematic representation of a double-mutant strain made with pTOP2S-EGFP-NEO5-APRT1i and confirmed by diagnostic PCR. PCR primer sets indicated by white triangles. (**B**) Fragments from TOP2S-EGFP and HHT-mCherry genomic loci were amplified from DNA isolated from 6mp-resistant cells, while only the fragment from the HHT-mCherry genomic locus could be amplified from the original HHT-mCherry-expressing cells. (**C**) Only the double mutant strain expressed TOPS-EGFP, which was localized exclusively to the MAC, and HHT-mCherry, which was localized to both the MAC and the MIC. Numerous food vacuoles dispersed in the cytoplasm had green autofluorescence. Scale bar denotes 10 µm.

After biolistic bombardment, the cells were incubated in SPP at 30 °C for 24 h followed by exposure to various concentrations of 6mp (0.47, 0.95, 1.9, 3.8, 7.5, and 15 μg/mL). For the RNAi-based system, we added 1 μg/mL CdCl_2_ to induce the production of short hairpin RNA against *APRT1*. Aliquots were then transferred to 96-well plates, which were incubated at 30 °C for 3 days. We found that >1.9 μg/mL concentrations of 6mp killed all cells within 3 days, whereas lower concentrations hardly affected cells. However, in 1.9 μg/mL 6mp a small fraction of cells in every well survived, and we observed slow propagation in some of these wells, probably due to the depletion of 6mp. We transferred 50-μL aliquots from these wells to new 96-well plates with 200 μL/well SPP plus 1.9 μg/mL 6mp. After incubation at 30 °C for 3 days, a single well from the RNAi-based system contained rapidly proliferating putative transformed cells. No such transformants were seen for the other *APRT1* locus-based systems, even after incubating them for another 5 days. Diagnostic PCR (Figure 9A,B) and fluorescence microscopic observation of live cells (Figure 9C) verified that the RNAi-based 6mp^r^ transformants were double mutant transformants. Due to the *NEO5* cassettes present in both the HHT-mCherry and pTOP2S-EGFP constructs (Figure 9A), the double transformants could be stably maintained even after cessation of RNAi by culturing the cells with paromomycin. We repeated the screening in triplicate, and the RNAi-based system consistently yielded double mutant transformants (Table 1), although the efficiency was not as high as with paromomycin- and puromycin-resistant systems [8,11]. We believe, however, that the system can be optimized by manipulating 6mp concentrations.

In *T. thermophila*, paromomycin is the most popular selection drug. It is used not only to select for *NEO*-based constructs, but also to select for autonomously replicating vectors carrying a mutation in the 17S rRNA gene [38], which makes cells resistant to paromomycin [39]. The *APTR1*-RNAi-*NEO5* hybrid system could have useful applications in creating double mutant transformants in paromomycin-resistant strains for the following reasons: (1) when cadmium is present, it provides a selection marker for 6mp, and (2) paromomycin alone maintains the two different transgenes after screening. Unfortunately, we found that the *APRT1* locus-based systems failed to give us transformants, no doubt because the acquisition of 6mp resistance requires a substantial reduction in the number of wild-type *APRT1* copies. The *APRT1* locus-based system could serve as a markerless transformation tool for gemline knockouts [28]. Transformation during meiotic prophase results in progeny MACs that have lost 50% of their wild-type *APRT1* copies, enough to allow them to become 6mp resistant (as heterozygotes are resistant). In this scenario, homozygous homokaryons (*MPR1-1*/*MPR1-1*, 6mp^s^) are a prerequisite for both parents.

## 4. Conclusions

Classical 6mp resistance in *T. thermophila* is due to inactivation of the APRTase by a mutation in the MIC *APRT1* gene. This knowledge may be used to create novel tools for genetic manipulation of *Tetrahymena*.

## Figures and Tables

**Table 1:** Number of wells with 6mp-resistant cells.

Transformation Plasmids	Exp. 1	Exp. 2	Exp. 3	Exp. 4
pD127N-EGFP-ATG8.2	0/96	0/96	0/96	0/96
pEGFP-ATG8.2 APRT1 locus	0/96	0/96	0/96	0/96
pTOP2S-EGFP-NEO5-APRT1i	1/96	0/96	1/96	1/96

## Supplementary Materials

The following are available online at http://www.mdpi.com/2073-4425/9/4/179/s1, Figure S1: Map of the endogenous APRTase expression plasmid pD127N-FZZ-PAC, with a puromycin resistance cassette (PAC), FZZ tag containing polyA signal, mutated *APRT1* ORF plus 5′ UTR, and 3′ UTR of *APRT1*., Figure S2: Map of the MAC *APRT1* knockout plasmid pΔAPRT1-NEO5, with paromomycin-resistance cassette (*NEO5*) and 5′ and 3′ flanking sequences of APRT1., Figure S3: Effect of overexpression of mutant APRTase on cell sensitivity to 6mp., Figure S4: Map of the RNAi backbone plasmid pAkRNAi-NEO5, with paromomycin resistance cassette (*NEO5*), MTT1 promoter, junk DNA sequences separated by a linker sequence, and 5′ and 3′ UTRs of BTU1., Figure S5: Map of the *APRT1* knockdown plasmid pAPRT1i-NEO5, with paromomycin resistance cassette (*NEO5*), cadmium-inducible MTT1 promoter, hairpin RNAi cassette, and 5′ and 3′ UTRs of the MAC BTU1., Figure S6: Map of trial plasmid pD127N-EGFP-ATG8.2., Figure S7: Map of trial plasmid pEGFP-ATG8.2 *APRT1* locus., Figure S8: Map of trial plasmid pTOP2S-EGFP-NEO5-APRT1i.
