# Supplementary material for: Resistance to 6-Methylpurine is Conferred by Defective Adenine Phosphoribosyltransferase in Tetrahymena
Source: Genes (Basel). 2018 Mar 23;9(4):179. doi: 10.3390/genes9040179 (PMC5924521; doi:10.3390/genes9040179)
Supplement: Supplementary file 1 [file genes-09-00179-s001.zip › Figure S8.pdf]

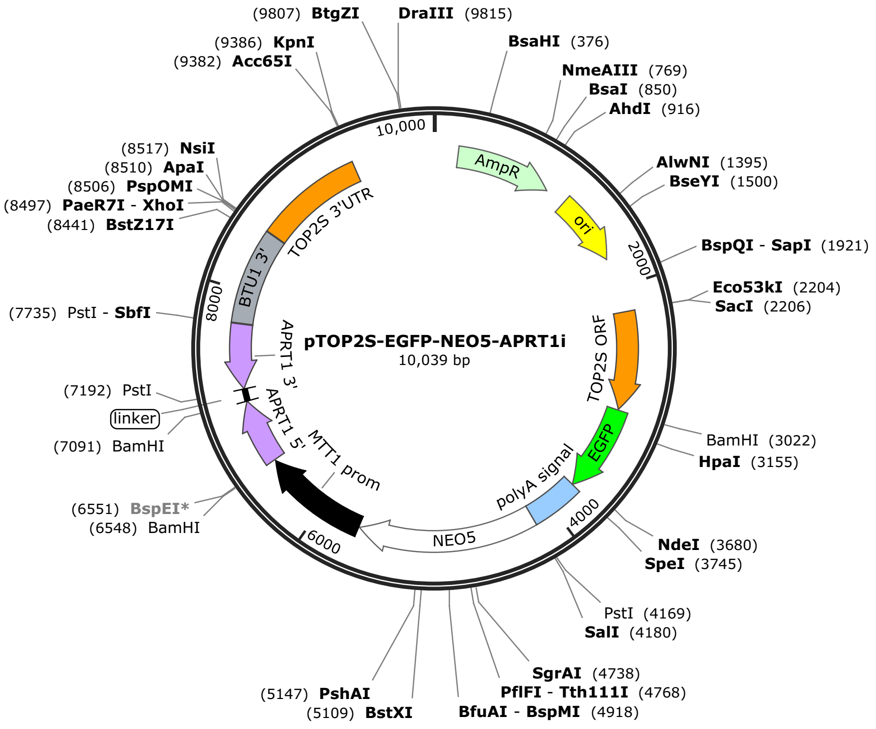

Figure S8. Map of trial plasmid pTOP2S-EGFP-NEO5-APRT1i. RNAi cassette and *NEO5* cassette were excised from pAPRT1i-NEO5 and then integrated into the SalI–ApaI sites of pTOP2S-EGFP-NEO4 [1].

ACGT: TOP2S ORF
acgt: TOP2S 3′ UTR
3′ UTR of BTU1
EGFP
polyA signal
ACGT: APRT1 CDS
NEO5 cassette
MTT1 promoter
Linker

GTGGCACTTTTCGGGGAAATGTGCGCGGAACCCCTATTTGTTTATTTTTCTAAATACATTCAAATA
TGTATCCGCTCATGAGACAATAACCCTGATAAATGCTTCAATAATATTGAAAAAGGAAGAGTATGA
GTATTCAACATTTCCGTGTCGCCCTTATTCCCTTTTTTGCGGCATTTTGCCTTCCTGTTTTTGCTC
ACCCAGAAACGCTGGTGAAAGTAAAAGATGCTGAAGATCAGTTGGGTGCACGAGTGGGTTACATCG
AACTGGATCTCAACAGCGGTAAGATCCTTGAGAGTTTTCGCCCCGAAGAACGTTTTCCAATGATGA
GCACTTTTAAAGTTCTGCTATGTGGCGCGGTATTATCCCGTATTGACGCCGGGCAAGAGCAACTCG
GTCGCCGCATACACTATTCTCAGAATGACTTGGTTGAGTACTCACCAGTCACAGAAAAGCATCTTA

CGGATGGCATGACAGTAAGAGAATTATGCAGTGCTGCCATAACCATGAGTGATAACACTGCGGCCA
ACTTACTTCTGACAACGATCGGAGGACCGAAGGAGCTAACCGCTTTTTTGCACAACATGGGGGATC
ATGTAACTCGCCTTGATCGTTGGGAACCGGAGCTGAATGAAGCCATACCAAACGACGAGCGTGACA
CCACGATGCCTGTAGCAATGGCAACAACGTTGCGCAAACTATTAACTGGCGAACTACTTACTCTAG
CTTCCCGGCAACAATTAATAGACTGGATGGAGGCGGATAAAGTTGCAGGACCACTTCTGCGCTCGG
CCCTTCCGGCTGGCTGGTTTATTGCTGATAAATCTGGAGCCGGTGAGCGTGGGTCTCGCGGTATCA
TTGCAGCACTGGGGCCAGATGGTAAGCCCTCCCGTATCGTAGTTATCTACACGACGGGGAGTCAGG
CAACTATGGATGAACGAAATAGACAGATCGCTGAGATAGGTGCCTCACTGATTAAGCATTGGTAAC
TGTCAGACCAAGTTTACTCATATATACTTTAGATTGATTTAAAACTTCATTTTTAATTTAAAAGGA
TCTAGGTGAAGATCCTTTTTGATAATCTCATGACCAAAATCCCTTAACGTGAGTTTTCGTTCCACT
GAGCGTCAGACCCCGTAGAAAAGATCAAAGGATCTTCTTGAGATCCTTTTTTTCTGCGCGTAATCT
GCTGCTTGCAAACAAAAAAACCACCGCTACCAGCGGTGGTTTGTTTGCCGGATCAAGAGCTACCAA
CTCTTTTTCCGAAGGTAACTGGCTTCAGCAGAGCGCAGATACCAAATACTGTCCTTCTAGTGTAGC
CGTAGTTAGGCCACCACTTCAAGAACTCTGTAGCACCGCCTACATACCTCGCTCTGCTAATCCTGT
TACCAGTGGCTGCTGCCAGTGGCGATAAGTCGTGTCTTACCGGGTTGGACTCAAGACGATAGTTAC
CGGATAAGGCGCAGCGGTCGGGCTGAACGGGGGGTTCGTGCACACAGCCCAGCTTGGAGCGAACGA
CCTACACCGAACTGAGATACCTACAGCGTGAGCTATGAGAAAGCGCCACGCTTCCCGAAGGGAGAA
AGGCGGACAGGTATCCGGTAAGCGGCAGGGTCGGAACAGGAGAGCGCACGAGGGAGCTTCCAGGGG
GAAACGCCTGGTATCTTTATAGTCCTGTCGGGTTTCGCCACCTCTGACTTGAGCGTCGATTTTTGT
GATGCTCGTCAGGGGGGCGGAGCCTATGGAAAAACGCCAGCAACGCGGCCTTTTTACGGTTCCTGG
CCTTTTGCTGGCCTTTTGCTCACATGTTCTTTCCTGCGTTATCCCCTGATTCTGTGGATAACCGTA
TTACCGCCTTTGAGTGAGCTGATACCGCTCGCCGCAGCCGAACGACCGAGCGCAGCGAGTCAGTGA
GCGAGGAAGCGGAAGAGCGCCCAATACGCAAACCGCCTCTCCCCGCGCGTTGGCCGATTCATTAAT
GCAGCTGGCACGACAGGTTTCCCGACTGGAAAGCGGGCAGTGAGCGCAACGCAATTAATGTGAGTT
AGCTCACTCATTAGGCACCCCAGGCTTTACACTTTATGCTTCCGGCTCGTATGTTGTGTGGAATTG
TGAGCGGATAACAATTTCACACAGGAAACAGCTATGACCATGATTACGCCAAGCGCGCAATTAACC
CTCACTAAAGGGAACAAAAGCTGGAGCTCACGCTAAGGAGCAGACCTCGAAAATGAAGAAGAGTAG
CTGACCAAAAAAAGAGTTCTGAATGAAGGAGAACCAAAAAGTATAGCTTAAAATAAAAAATAAACA
AAAGAAATAAATGATGAAGATCTTGAATATAATTTGTTTAAGGATGAAAAGCCAAAGTAAAAAAAG
CCTCCTGCTACTGCTGCTTCAAAATCAAAGAAAGTGATAAATAAAAATGAAGATGATGATGAAGAC
TTTAATGATGATTTATCAGAAGATGAAAAACCAAAGAAGAAAAGGTCCAATTAAACTTAATCAAAA
ACCAAAAAATAGAAAAATGAAAGTGAAGATGAAGATTTTAATAGCAAAGAATCTGATGAAAAACCT
AAATAAAGAAAATCTAATTCTAACTCATAAAAAGCAAGGAAACCTAAGAAAATGAGTGATGATGAG
GGAGAAGAAGATGATGATTTTATCATACCTGATGAGGATTCTGATGAAGAAAAACCTAAGAAGAGA
AAGAGTAATGCAACAAAACCTAAAAATAATAAAGAAGATAAAGATAATCAAAAGTCTAAAAAAAAT
AGAACCGATTCACCAAAACTTAAAATAACAAATTCAGAAGATGAAGATGTTGCTGAATTAAAGCAA
AATGAAGTTTTATAAACCAGAACAAGAGGTAAAAGAAACCCAATTCCTCGTAAAATGATCATTGAT
AATTCTGATGAAGATTTCAGTGAAGAGGAAGAATTTGGAAATAGCAAAAAGAAATCAGTAAATAGT
AAAAGCCAAAAAAAATAAAAAGACGATGAATCATCGGATGAATGCTATTTCGGATCCATGGTTTCT
AAGGGTGAAGAACTTTTCACTGGTGTTGTTCCTATTTTAGTTGAATTAGATGGTGATGTTAATGGT
CACAAATTTTCTGTTTCAGGTGAAGGTGAAGGTGATGCTACTTATGGTAAGTTAACTCTTAAATTT
ATTTGTACTACTGGTAAGTTACCTGTTCCTTGGCCTACTTTAGTTACTACTCTTACTTATGGTGTT
CAATGCTTTTCAAGATACCCTGATCACATGAAGCAACATGATTTCTTTAAGTCTGCTATGCCTGAA
GGTTATGTTCAAGAAAGAACTATTTTCTTTAAAGATGATGGTAATTATAAAACTAGAGCTGAAGTT

AAATTTGAAGGTGATACTTTAGTTAATAGAATTGAACTTAAAGGTATTGATTTCAAGGAAGATGGT
AATATTCTTGGTCATAAACTTGAATATAATTACAATTCTCATAATGTTTACATTATGGCTGATAAG
CAAAAGAATGGTATTAAAGTTAATTTTAAAATTAGACATAATATTGAAGATGGTTCAGTTCAATTA
GCTGATCATTATCAACAAAATACTCCTATTGGTGATGGTCCTGTTCTTTTACCTGATAATCACTAC
TTATCTACTCAATCAGCTTTATCTAAAGATCCTAATGAAAAAAGAGATCATATGGTTCTTTTAGAA
TTTGTTACTGCTGCTGGTATTACTCTTGGTATGGATGAATTATATAAGACTAGTTGAGCGAACTGA
ATCGGTCAGCTAAACCAACCAATCAACATAATAAACTTTATTATTTTTACTTAAGCATCTTACTGT
TGTTGTAATAGTAGAGAAAGAAATACCCAATTAACTTCATTCACATAACATTAATATCTATAAACA
TCTTTTTTCTCACATATATACAACTCTCTAAATCAACAAATAACTTTTTAAAAATAATGGATATAT
ATTAACAAAATAATATATCTCTTTTTACAAAATAGTTCTTATATAAATACGTATTCTGCACTCACC
CGCATTTTTCACAACAAAAACATACCAAAAAAATTCTTACTTCTACATGTTTCCTTTCTTATTATT
ACAAAATTATTTTATAAATAGCATACAAAAATAAATACAATAAAAAAATAAACAAAATCCAGATCC
CCCGGGCTGCAGGAATTCGATGTCGACTTGATATCTTCAAAGTATGGATTAATTATTTCAAATTAT
TAGAAGGTAATTAATCTGCATAAATTCAAAACTATAAAAATAAAACATTAAAATTAATTCAACCTT
ATTGAAGCATCAAAATCTGAATCTCTAGAAAGACTGATTCTGATTGGATAATTTTTCGGCGCTAAG
GATTTTGGATTAAAGAAAATTAGATTTAATTATTAATCATGATTTGAATAGGATAGCAAGAATATT
TGTTTGGTTTAAAAGGGAAAGCGGGTAATTATCAAAAATTTATAAATAATTTTAAAACAATAAATA
GAAAAACAAATAAGATTATAAAAACTTACAAAAATGATTGAACAAGATGGTTTACACGCTGGTTCT
CCCGCCGCTTGGGTCGAAAGACTTTTCGGTTATGACTGGGCTCAACAAACCATCGGTTGCTCTGAT
GCCGCCGTCTTCCGTCTTTCTGCTCAAGGTCGTCCTGTTCTTTTCGTCAAGACCGACCTTTCTGGT
GCCCTTAATGAACTTCAAGATGAAGCTGCCCGTCTTTCTTGGCTTGCCACCACCGGTGTTCCTTGC
GCTGCTGTCCTTGACGTTGTCACTGAAGCCGGTAGAGACTGGCTTCTTTTAGGTGAAGTCCCCGGT
CGAGATCTTCTTTCTTCTCACCTTGCTCCTGCCGAAAAAGTTTCTATCATGGCTGATGCTATGCGT
CGTCTTCATACCCTTGATCCCGCTACCTGCCCTTTCGACCACCAAGCCAAACATCGTATCGAACGT
GCTCGTACTCGTATGGAAGCCGGTCTTGTCGATCAAGATGATCTTGACGAAGAACATCAAGGTCTT
GCCCCTGCCGAACTTTTCGCCAGACTTAAGGCCCGTATGCCCGACGGTGAAGATCTTGTCGTCACC
CATGGTGATGCCTGCTTACCCAATATCATGGTTGAAAATGGTCGTTTTTCTGGTTTCATCGACTGT
GGTCGTCTTGGTGTCGCCGACCGTTATCAAGATATTGCCTTAGCTACCCGTGATATCGCTGAAGAA
CTTGGTGGTGAATGGGCTGACCGTTTCCTTGTCCTTTACGGTATCGCCGCTCCCGATTCTCAACGT
ATCGCCTTCTATCGTCTTCTTGACGAATTCTTCTGAGATCCTTAAATTAAAAATTCAATATATATT
TACAAACTTTCATATAAAATAAATATATTATATAAAATTAATTTTTAGTGTATTATATTAACATTA
AAGCACCAAAAAAACGTGTTAATATACTACTATAAAATATAATTTATTCCAAATTGACTAAAATCA
TTATTTTACAACTCATTTGTATATATATATTTTATGTCAATTATTTTTTTTAACTTTCTAAAAAAAAA
AATTCCTCTTCACATACATGTTAGCTCTTAAAAATTTGTCTGCAAATCCAATAATAATATTTTTTT
TTGCCATTAAATTTTCAAATTTTTACTGGAAAAATGCAGCCCGGGGGATCAGACAATTTATTTCTA
AAAAATATTTAAAAATAAAAAATAATAAGGGTTTTGAATAACTCCTTTAATTTAAATACACATTTT
TAAATTTTTTTTAGCTCTTTAAATATTCATAAAAATAAAAAATAACTAACTAAAAATAAATAAAAA
GATAATAATGATTAAAGGTATAATACTGAATAAGAAAAAACATAATAGAGTACTTATTTTTTATAT
CACTATTTTTAATATCTTGAAAGCAAAACTTTTTTATATATCTTAAAATATATTGTATCGTTTATT
CAATTATTTTCTTTAAATTTCAAATATATTGATAAAAAAGATGACATGTTTTTTAAAGAAAACATG
AAATATAAAATAGATAAATATCAATTATTTTATTTATTAAATATATAAGCTGCTCAAAACATAGCT
CATTCATCAATTATAATATGTGAATCATTAATTTTCAAAATATTACTCATTATTTAGGCTATCATT
TATTTTTTATTTTCAATTATCCGTTTCTATTATATTTTAATATTAAGTTGTGATTCTTGAATTTTG
TGTCATGAATTATTTGTAAATCTTTTTATTTCTGATAAAAAATATAAATTGATTGACTCATGATTT

AAATCATGAGTCAACCTAACTAATTTTCAAAATTCTTCTATTCTAAAATATAGATGTGATTCTTGA
ATCTCTCTTGAATATAAAGTAATTTTTTATATTTCTGATATAATTCTTAGCTACGTGATTCACGAT
TTATGCAATGATCCATATAAAATAATGTAAATAGTGTATATATATATATTCGTCTTTTTTATTCTT
TATATAATTTAAAAAAATTAAAAAAATTTAATAAAGCTCTAATAAAATAAATAATAATACTAAACT
TAAACATCCCGGGGGATCCGGATTACTAAAATTAAGAAATAGGTAAAGTTATTAGCGAAAACTTCA
GAAATATTCCTGATTTCCCTTAACCTGGTATTATCTTTAAAGATATTGCTCCTCTCCTCTCTAATC
CTGCCATTTTTAAGCGTATTACTGATGCTTTTGTAATTGCTTTGAAGGACATTGATTTTGATAAAA
TTGTTATCTTAGAATCTAGAGGTTTTATTTTTGGAACCGCTATTGGTTTACAAATGGGAAAGGGAT
TAATTTTAATCAGAAAGAAAGGCAAACTACCTGGTAAGGTTGAATAATTCTCATTCGACTTAGAAT
ACGGTAAGGATGTTTTTGAAATTCAAAGTGATGCTTTAGCTAAAGGTGAAAAAGTTGTTATTGTTG
ATGATATTCTAGCTACTGGTGGAACTGCTGATGCAGCTTGCAGATTAGTTGAAAAGGTAGGAGGAG
TAGTATAAGCTGTTATGTTCCTTTACCAAATTAAGGATCTTAAGGGCGAAGAAAGATTATCTAAAT
ACAAGGTTATTAATCTTCATATTGAATG**GGATCCGTAAGTAACGGATATGCAAAAGtaaacaatt**
ttaggcttttataaaaaataggaagcaggccggcggTAGATAAAAGAAGAATTCAAAGGCTGCAGC
ATTCAATATGAAGATTAATAACCTTGTATTTAGATAATCTTTCTTCGCCCTTAAGATCCTTAATTT
GGTAAAGGAACATAACAGCTTATACTACTCCTCCTACCTTTTCAACTAATCTGCAAGCTGCATCAG
CAGTTCCACCAGTAGCTAGAATATCATCAACAATAACAACTTTTTCACCTTTAGCTAAAGCATCAC
TTTGAATTTCAAAAACATCCTTACCGTATTCTAAGTCGAATGAGAATTATTCAACCTTACCAGGTA
GTTTGCCTTTCTTTCTGATTAAAATTAATCCCTTTCCCATTTGTAAACCAATAGCGGTTCCAAAAA
TAAAACCTCTAGATTCTAAGATAACAATTTTATCAAAATCAATGTCCTTCAAAGCAATTACAAAAG
CATCAGTAATACGCTTAAAAATGGCAGGATTAGAGAGGAGAGGAGCAATATCTTTAAAGATAATAC
CAGGTTAAGGGAAATCAGGAATATTTCTGAAGTTTTCGCTAATAACTTTACCTATTTCTTAATTTT
AGTAATCCCTGCAGGCGAACTGAATCGGTCAGCTAAACCAACCAATCAACATAATAAACTTTATTA
TTTTTACTTAAGCATCTTACTGTTGTTGTAATAGTAGAGAAAGAAATACCCAATTAACTTCATTCA
CATAACATTAATATCTATAAACATCTTTTTTCTCACATATATACAACTCTCTAAATCAACAAATAA
CTTTTTAAAAATAATGGATATATATTAACAAAATAATATATCTCTTTTTACAAAATAGTTCTTATA
TAAATACGTATTCTGCACTCACCCGCATTTTTCACAACAAAAACATACCAAAAAAATTCTTACTTC
TACATGTTTCCTTTCTTATTATTACAAAATTATTTTATAAATAGCATACAAAAATAAATACAATAA
AAAAATAAACAAAATCCTTTTTTATTTTGAATTATTTAAAACAAATATTTTCAATCAATCAGTCAG
TCAGCATAATATTAAAGCAACAAAACAAACCCAAGTTGTTTTTATAGTTTTTTAATTGCTTTTCAG
TACTATAAATAAATTTGTTATTACTTCAAGATTGATAAACTTCTTTTTTAAATTAAATATCTATGA
ATGAATAAATAAGTTGATATCTCTTTTTAACTTGTTTTCCTCTCTTTTACTTACTTGCCAATTTTT
TTTTTAAATTAAAGAAATATCTTTTTATTTTTCAAAAACAAAATTTATTTTCCCTTGTATACAAAA
ACCCCTTTATTTAAATAAAATCTTTATGCCCATCAATAGCCACATCTTCTCGAGGGGGGGCCCcat
gcattcattcaatcaatcaatcacactcagctttaaataatttaaaaagatattccgcaaatttta
gaatttaaagttggtaaaaaaagttatcaaatttgcaatatttctattgatttatatatttatcaa
tcagtcaatgctgacgatgtaatttaaaagatgtgttgtttttttctagattatttaactaattatt
aatatttaagaaatttttaaaaattttgaaatttattaaaagtttaaaatttgttaaaaaataatt
aaaaaattaaagtaaaagaaaaaataaaaatgatagacaattagaatataggaagtgacgacgaat
tagatatttatgaaccaggaacttcaggcgtatgaaaatattttatatagtttgtattgtaatagg
ttaaataagatatagcttaaattctttcaattctaaaataggaaatcattagctacttgaaaagaa
actttaatacattaaatgattaggttttaggagctagaaaaaaagctatcctcaacatttatgaaa
aactatctgaaagctaatttgctatttctggaagtaaaaattatctctttagaaactcaaatcaag
ctaaattatttcattttttgcaaatatttaaagtggtttctcaatagatcatggttaagttcaataa

ataattattataatgtttttcggatagagcagagaagattagagaatactctatcaaaattatttc
tgagtaattttttattaattttaattgctatgtttttaaataaatgttttgaattcagattattgt
ccaaatgtgatgatatgtaacctttcttaccttatgtgatatctattgtttgtgagagagttaatt
gccaagaccGGTACCCAATTCGCCCTATAGTGAGTCGTATTACGCGCGCTCACTGGCCGTCGTTTT
ACAACGTCGTGACTGGGAAAACCCTGGCGTTACCCAACTTAATCGCCTTGCAGCACATCCCCCTTT
CGCCAGCTGGCGTAATAGCGAAGAGGCCCGCACCGATCGCCCTTCCCAACAGTTGCGCAGCCTGAA
TGGCGAATGGGACGCGCCCTGTAGCGGCGCATTAAGCGCGGCGGGTGTGGTGGTTACGCGCAGCGT
GACCGCTACACTTGCCAGCGCCCTAGCGCCCGCTCCTTTCGCTTTCTTCCCTTCCTTTCTCGCCAC
GTTCGCCGGCTTTCCCCGTCAAGCTCTAAATCGGGGGCTCCCTTTAGGGTTCCGATTTAGTGCTTT
ACGGCACCTCGACCCCAAAAAACTTGATTAGGGTGATGGTTCACGTAGTGGGCCATCGCCCTGATA
GACGGTTTTTCGCCCTTTGACGTTGGAGTCCACGTTCTTTAATAGTGGACTCTTGTTCCAAACTGG
AACAACACTCAACCCTATCTCGGTCTATTCTTTTGATTTATAAGGGATTTTGCCGATTTCGGCCTA
TTGGTTAAAAAATGAGCTGATTTAACAAAAATTTAACGCGAATTTTAACAAAATATTAACGCTTAC
AATTTAG

References

1. Akematsu, T.; Fukuda, Y.; Garg, J.; Fillingham, J. S.; Pearlman, R. E.; Loidl, J. Post-meiotic DNA double-strand breaks occur in Tetrahymena, and require Topoisomerase II and Spo11. *eLife* **2017**, *6*, e26176, doi:10.7554/eLife.26176.
