# Supplementary material for: Resistance to 6-Methylpurine is Conferred by Defective Adenine Phosphoribosyltransferase in Tetrahymena
Source: Genes (Basel). 2018 Mar 23;9(4):179. doi: 10.3390/genes9040179 (PMC5924521; doi:10.3390/genes9040179)
Supplement: Supplementary file 1 [file genes-09-00179-s001.zip › Figure S1.pdf]

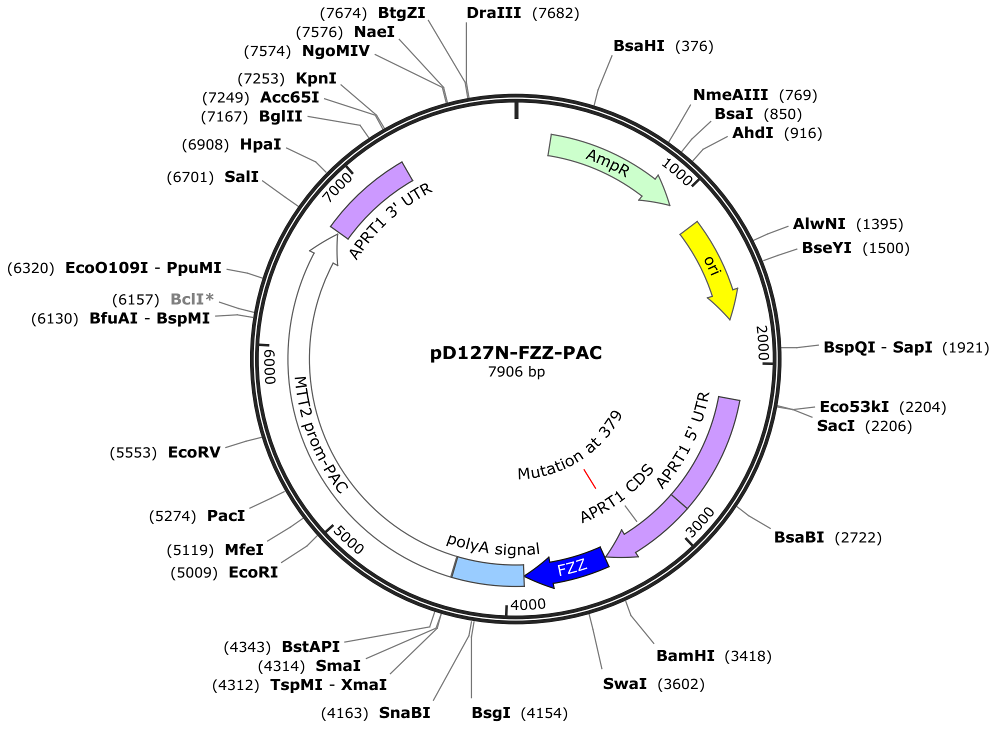

Figure S1. Map of the endogenous APRTase expression plasmid pD127N-FZZ-PAC, with a puromycin resistance cassette (*PAC*), FZZ tag containing polyA signal, mutated *APRT1* ORF plus 5′ UTR, and 3′ UTR of *APRT1*. Control plasmid carries wild-type *APRT1* instead of the mutated version.

acgt: 5′ and 3′ UTRs of APRT1

ACGT: APRT1 CDS

A: Mutation at 379

FZZ tag

polyA signal

MTT2 promoter-PAC cassette

GTGGCACTTTTCGGGGAAATGTGCGCGGAACCCCTATTTGTTTATTTTTCTAAATACATTCAAATA
TGTATCCGCTCATGAGACAATAACCCTGATAAATGCTTCAATAATATTGAAAAAGGAAGAGTATGA
GTATTCAACATTTCCGTGTCGCCCTTATTCCCTTTTTTGCGGCATTTTGCCTTCCTGTTTTTGCTC
ACCCAGAAACGCTGGTGAAAGTAAAAGATGCTGAAGATCAGTTGGGTGCACGAGTGGGTTACATCG
AACTGGATCTCAACAGCGGTAAGATCCTTGAGAGTTTTCGCCCCGAAGAACGTTTTCCAATGATGA
GCACTTTTAAAGTTCTGCTATGTGGCGCGGTATTATCCCGTATTGACGCCGGGCAAGAGCAACTCG
GTCGCCGCATACACTATTCTCAGAATGACTTGGTTGAGTACTCACCAGTCACAGAAAAGCATCTTA
CGGATGGCATGACAGTAAGAGAATTATGCAGTGCTGCCATAACCATGAGTGATAACACTGCGGCCA
ACTTACTTCTGACAACGATCGGAGGACCGAAGGAGCTAACCGCTTTTTTGCACAACATGGGGGATC

ATGTAACTCGCCTTGATCGTTGGGAACCGGAGCTGAATGAAGCCATACCAAACGACGAGCGTGACA
CCACGATGCCTGTAGCAATGGCAACAACGTTGCGCAAACTATTAACTGGCGAACTACTTACTCTAG
CTTCCCGGCAACAATTAATAGACTGGATGGAGGCGGATAAAGTTGCAGGACCACTTCTGCGCTCGG
CCCTTCCGGCTGGCTGGTTTATTGCTGATAAATCTGGAGCCGGTGAGCGTGGGTCTCGCGGTATCA
TTGCAGCACTGGGGCCAGATGGTAAGCCCTCCCGTATCGTAGTTATCTACACGACGGGGAGTCAGG
CAACTATGGATGAACGAAATAGACAGATCGCTGAGATAGGTGCCTCACTGATTAAGCATTGGTAAC
TGTCAGACCAAGTTTACTCATATATACTTTAGATTGATTTAAAACTTCATTTTTAATTTAAAAGGA
TCTAGGTGAAGATCCTTTTTGATAATCTCATGACCAAAATCCCTTAACGTGAGTTTTCGTTCCACT
GAGCGTCAGACCCCGTAGAAAAGATCAAAGGATCTTCTTGAGATCCTTTTTTTCTGCGCGTAATCT
GCTGCTTGCAAACAAAAAAACCACCGCTACCAGCGGTGGTTTGTTTGCCGGATCAAGAGCTACCAA
CTCTTTTTCCGAAGGTAACTGGCTTCAGCAGAGCGCAGATACCAAATACTGTCCTTCTAGTGTAGC
CGTAGTTAGGCCACCACTTCAAGAACTCTGTAGCACCGCCTACATACCTCGCTCTGCTAATCCTGT
TACCAGTGGCTGCTGCCAGTGGCGATAAGTCGTGTCTTACCGGGTTGGACTCAAGACGATAGTTAC
CGGATAAGGCGCAGCGGTCGGGCTGAACGGGGGGTTCGTGCACACAGCCCAGCTTGGAGCGAACGA
CCTACACCGAACTGAGATACCTACAGCGTGAGCTATGAGAAAGCGCCACGCTTCCCGAAGGGAGAA
AGGCGGACAGGTATCCGGTAAGCGGCAGGGTCGGAACAGGAGAGCGCACGAGGGAGCTTCCAGGGG
GAAACGCCTGGTATCTTTATAGTCCTGTCGGGTTTCGCCACCTCTGACTTGAGCGTCGATTTTTGT
GATGCTCGTCAGGGGGGCGGAGCCTATGGAAAAACGCCAGCAACGCGGCCTTTTTACGGTTCCTGG
CCTTTTGCTGGCCTTTTGCTCACATGTTCTTTCCTGCGTTATCCCCTGATTCTGTGGATAACCGTA
TTACCGCCTTTGAGTGAGCTGATACCGCTCGCCGCAGCCGAACGACCGAGCGCAGCGAGTCAGTGA
GCGAGGAAGCGGAAGAGCGCCCAATACGCAAACCGCCTCTCCCCGCGCGTTGGCCGATTCATTAAT
GCAGCTGGCACGACAGGTTTCCCGACTGGAAAGCGGGCAGTGAGCGCAACGCAATTAATGTGAGTT
AGCTCACTCATTAGGCACCCCAGGCTTTACACTTTATGCTTCCGGCTCGTATGTTGTGTGGAATTG
TGAGCGGATAACAATTTCACACAGGAAACAGCTATGACCATGATTACGCCAAGCGCGCAATTAACC
CTCACTAAAGGGAACAAAAGCTGGAGCTCgagctgaatgaatgaatgaatgaatgtatttatgaat
gaattaatgcaataaataataaaataatactttttataatatttagtttaaaattatatgtctact
atatataaatattctttaaactccatcttaaagacaccaaaataccttagctatttattaggtttt
ataacaaaaatcatttgatttttttagttaagcaaacaagtaagcaatggttttaaatacaataaat
aattttatttatcttaaaatcaaattttattgaagatttttttattcaaataaattttctttattat
tcgttttgagtattttatcatcctcgcttactgattttgttcaaaatatccttttattgatattaa
ttgcttaaattaattagagttaagctcaatattttgagtgaaatttgaaaaattctgaaattattt
tcatttggaatttgcttttcaagatattctattttgtaataaaaataaaaatattaagacaattaa
ttagaaaaatagataaatatcactctattttcaggatttgctttactttctttttgattgatttgt
ggggtaataaaattaggataactaagtctgcttaattttaataaaataaataattattgatttaca
aagatattctcttaaaaaaataaattatagatagaagtgaaaATGGATTACTAAAATTAAGAAATA
GGTAAAGTTATTAGCGAAAACTTCAGAAATATTCCTGATTTCCCTTAACCTGGTATTATCTTTAAA
GATATTGCTCCTCTCCTCTCTAATCCTGCCATTTTTAAGCGTATTACTGATGCTTTTGTAATTGCT
TTGAAGGACATTGATTTTGATAAAATTGTTATCTTAGAATCTAGAGGTTTTATTTTTGGAACCGCT
ATTGGTTTACAAATGGGAAAGGGATTAATTTTAATCAGAAAGAAAGGCAAACTACCTGGTAAGGTT
GAATAATTCTCATTCGACTTAGAATACGGTAAGGATGTTTTTGAAATTCAAAGTGATGCTTTAGCT
AAAGGTGAAAAAGTTGTTATTGTTAATGATATTCTAGCTACTGGTGGAACTGCTGATGCAGCTTGC
AGATTAGTTGAAAAGGTAGGAGGAGTAGTATAAGCTGTTATGTTCCTTTACCAAATTAAGGATCTT
AAGGGCGAAGAAAGATTATCTAAATACAAGGTTATTAATCTTCATATTGAAGGATCCGATTACAAA
GATCATGATGGTGATTACAAAGATCACGATATTGATTACAAAGATGATGATGATAAAGCTGAAAAT

CTTTATTTTCAAGGTGCTTCTGCTCAACATGATGAAGCTGTTGATAACAAATTTAATAAGGAACAA
CAAAATGCTTTTTACGAAATTTTACATCTTCCTAATTTAAATGAAGAACAAAGAAATGCTTTCATT
CAATCATTAAAAGATGATCCTTCACAATCTGCTAATTTATTAGCTGAAGCTAAAAAATTAAATGAT
GCTCAAGCTCCTAAAGTTGATAATAAATTCAACAAAGAACAACAAAACGCTTTTTATGAAATTCTT
CATTTACCTAACCTTAATGAAGAACAAAGAAACGCTTTCATTCAATCTTTAAAGGATGATCCTTCT
CAATCTGCTAATTTACTTGCTGAAGCTAAAAAGTTAAATGGTGCTCAAGCTCCTAAGGTTGATGCT
AATACTAGTTGAGCGAACTGAATCGGTCAGCTAAACCAACCAATCAACATAATAAACTTTATTATT
TTTACTTAAGCATCTTACTGTTGTTGTAATAGTAGAGAAAGAAATACCCAATTAACTTCATTCACA
TAACATTAATATCTATAAACATCTTTTTTCTCACATATATACAACTCTCTAAATCAACAAATAACT
TTTTAAAAATAATGGATATATATTAACAAAATAATATATCTCTTTTTACAAAATAGTTCTTATATA
AATACGTATTCTGCACTCACCCGCATTTTTCACAACAAAAACATACCAAAAAAATTCTTACTTCTA
CATGTTTCCTTTCTTATTATTACAAAATTATTTTATAAATAGCATACAAAAATAAATACAATAAAA
AAATAAACAAAATCCAGATCCCCCGGGAAGCTTTCTAACTGTAAATGCAATCCTTGCAAATGTGCT
GAATGCAAATGCTGATTAATCAATATTGAATTTAAACAATAAATAAATAATTAAATCTATTTATTA
TATAATAACTTAAAAATAATATCTTAACCAAATCTAAAGTATGTATTGAATATATTTTATTTAGAA
TACATAATATAAATCTATATTTTAATTGTATCCTTATTTTTTTGACTTTTTAACATCTCAACTTAT
TCATTATATATTTTGCTTGCATTTGCTATTTCTAATCAACAACATAACATAAATTTATTACATCTT
GTTATTTTCATACATTCATTTTTGTTTTTTGATAGCAATAAAATATGTTTTTGTAATTTATCTAAA
AAATGAACAAAATAAATAAATTATTTTTGTATATTTTACACTATAGACGAGCAGCGAAGTAACTAA
ATACCATCAAATAATATTTTTAAAATGATTTAATTTTAATATGATATATTTATCATCAGTTTAATA
ACATTATTTAATCATTTCTAATCATTAAATATTGTTCAAAACAACTAACTAATTTACATGATTTTG
CAATCAACTCAATCTTTTATTTCATATAACATACACTAAAATATGAACTCGTATTTCTTTAGAAAT
TTTACATTTAAAAAATGTGATATATTTAACATTTTATGTGATTCGTTCATTTATTAGTGAATTCTA
CTTATTATACTTTTGTTTGATTAAATTGAAAACTCTTGAACTTTAAGAAGAAATTCAGATGATATT
TCTTAAGTAGATAAACAAATTTTTTAGAAAATCCTCCAATTGAATTAGTTTATTTAGGAAAGATAT
TAAATCAAATTCATATATGATATAAAAAAGAAAAAAACTCTTGAGCCTCATTTTTAGTTTATTTTA
CTTTGAAATAGAAATCTATTTTAAATCTTTATTGAAAATAATAAAGTGGTTAAACTTAATTAAATA
CTTACAAAAGAGCTTATAAGAATTAGTAAATAAATTCTACTAAAAATGGCTATATATTTATATCAA
AAACAGTTGTTTGCTTATTAAAACCTCCTTTATAATTTTTGACATATTTTTTTTATCAACAATAAG
TACTAAAAAAAATAACTTCATAAAATGTCTGATTTGCTTTTTTTTTTCTAGATCATAAATAGAATT
AAAATATAAAAAAATAACAAACATAAAATTACATAAATTTAATTACAGATAATTGTTTGATTGTAA
CAAAAAGATATCAGTAATTGTTAAAGTTAATCTGTAATACGAGAGTGATTCCTGAGGCTCAAGAGT
TTTTTAGAAAAATCTGATTAATTTTAATACAAATTTGCATATATAAATAATAGACCATTAACCCTA
ATTATCATAATCAAAAATAATTAACTAAAAAAATAACAAAAAAACAACTTTAAAAAAAAAAAACAAA
CAAAACAACTATAAAATAAATGACTGAATATAAGCCTACTGTTAGATTAGCTACTAGAGATGATGT
TCCTAGAGCTGTTAGAACTTTAGCTGCTGCTTTCGCTGATTATCCTGCTACTAGACACACTGTTGA
TCCTGATAGACACATTGAAAGAGTTACTGAATTATAAGAATTATTCTTAACTAGAGTTGGTTTAGA
TATTGGTAAGGTTTGGGTTGCTGATGATGGTGCTGCTGTTGCTGTTTGGACTACTCCTGAATCTGT
TGAAGCTGGTGCTGTTTTCGCTGAAATTGGTCCTAGAATGGCTGAATTATCTGGTTCTAGATTAGC
TGCTTAATAATAAATGGAAGGTTTATTAGCTCCTCACAGACCTAAGGAACCTGCTTGGTTCTTAGC
TACTGTTGGTGTTTCTCCTGATCACTAAGGTAAGGGTTTAGGTTCTGCTGTTGTTTTACCTGGTGT
TGAAGCTGCTGAAAGAGCTGGTGTTCCTGCTTTCTTAGAAACTTCTGCTCCTAGAAATTTACCTTT
CTATGAAAGATTAGGTTTCACTGTTACTGCTGATGTTGAAGTTCCTGAAGGTCCTAGAACTTGGTG
TATGACTAGAAAGCCTGGTGCTTGAGATCCTTAAATTAAAAATTCAATATATATTTACAAACTTTC

ATATAAAATAAATATATTATATAAAATTAATTTTTAGTGTATTATATTAACATTAAAGCACCAAAA
AAACGTGTTAATATACTACTATAAAATATAATTTATTCCAAATTGACTAAAATCATTATTTTACAA
CTCATTTGTATATATATTTTATGTCAATTATTTTTTTTAACTTTCTAAAAAAAAAAATTCCTCTTC
ACATACATGTTAGCTCTTAAAAATTTGTCTGCAAATCCAATAATAATATTTTTTTTTGCCATTAAA
TTTTCAAATTTTTACTGGAAAAATGCAGAAGCTTGTCGACCTCGAGgaatgaacaagttagtttgt
ttttgtatgaatgtgttttatatttatggatatagtaggaattaaatttttttgatttagtaattt
agtatgtacttcttaaagtttaaataataagatttgcattcaatttttgtaaattaagtagtttta
tgtttttttaactacataagtatttatcattgaatgtaattagttaactaaaatttttttatttcaac
atttttaaagaaagatttttactctttttgttgttatttatttattgttaaaaaggcttattaaaa
tagattgaattataaacaaaaagaataattaaaaaacagaatgaataaatgatctttctatatata
taaatatttttaattttcattaaattttataataaaaaatttatgatattattaattgactagtta
gctggtatcgtatatatatcctgcttttaaggaagtctagatctattaaattttaatttaccttag
catgaggttacttaaatatttatttgataggatgtcaaaggtcataaataaacgGGTACCCAATTC
GCCCTATAGTGAGTCGTATTACGCGCGCTCACTGGCCGTCGTTTTACAACGTCGTGACTGGGAAAA
CCCTGGCGTTACCCAACTTAATCGCCTTGCAGCACATCCCCCTTTCGCCAGCTGGCGTAATAGCGA
AGAGGCCCGCACCGATCGCCCTTCCCAACAGTTGCGCAGCCTGAATGGCGAATGGGACGCGCCCTG
TAGCGGCGCATTAAGCGCGGCGGGTGTGGTGGTTACGCGCAGCGTGACCGCTACACTTGCCAGCGC
CCTAGCGCCCGCTCCTTTCGCTTTCTTCCCTTCCTTTCTCGCCACGTTCGCCGGCTTTCCCCGTCA
AGCTCTAAATCGGGGGCTCCCTTTAGGGTTCCGATTTAGTGCTTTACGGCACCTCGACCCCAAAAA
ACTTGATTAGGGTGATGGTTCACGTAGTGGGCCATCGCCCTGATAGACGGTTTTTCGCCCTTTGAC
GTTGGAGTCCACGTTCTTTAATAGTGGACTCTTGTTCCAAACTGGAACAACACTCAACCCTATCTC
GGTCTATTCTTTTGATTTATAAGGATTTTGCCGATTTCGGCCTATTGGTTAAAAAATGAGCTGAT
TTAACAAAAATTTAACGCGAATTTTAACAAAATATTAACGCTTACAATTTAG
