# Supplementary material for: Resistance to 6-Methylpurine is Conferred by Defective Adenine Phosphoribosyltransferase in Tetrahymena
Source: Genes (Basel). 2018 Mar 23;9(4):179. doi: 10.3390/genes9040179 (PMC5924521; doi:10.3390/genes9040179)
Supplement: Supplementary file 1 [file genes-09-00179-s001.zip › Figure S3.pdf]

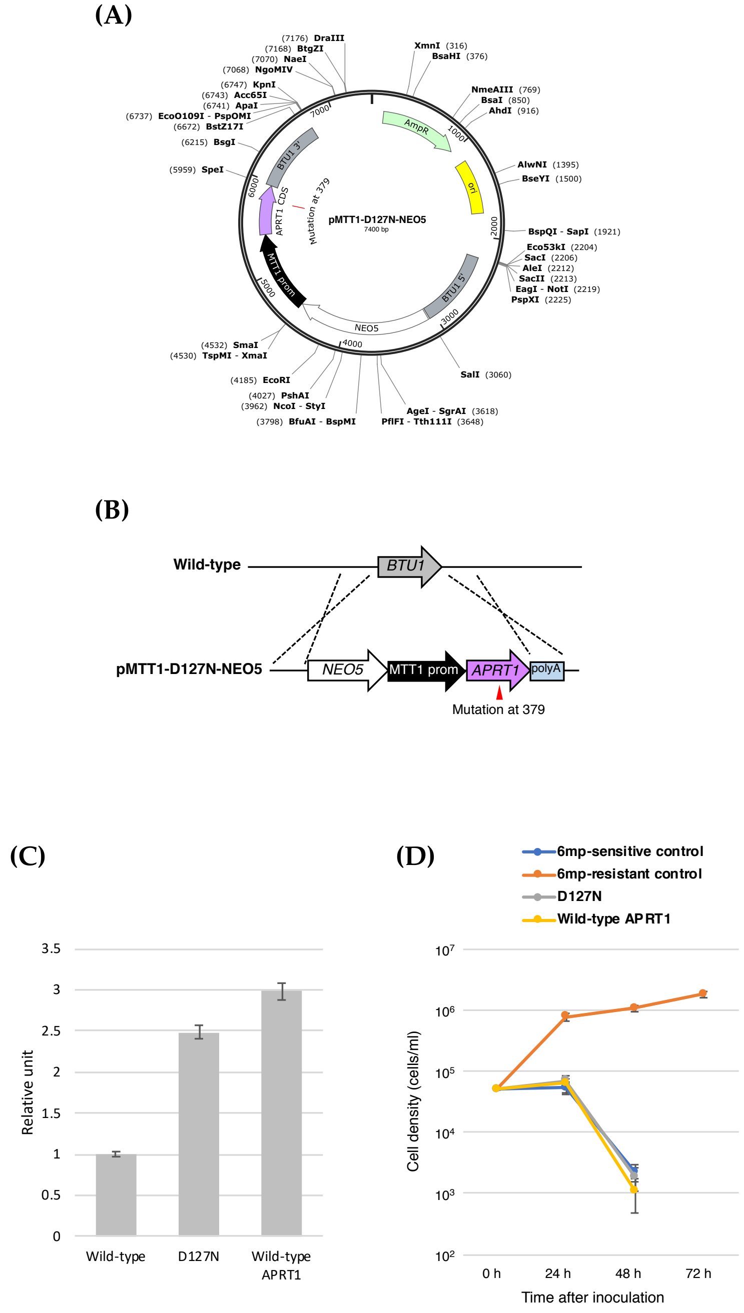

Figure S3. Effect of overexpression of mutant APRTase on cell sensitivity to 6mp. The backbone of pBNMB1-EGFP (a gift from Dr. Kazufumi Mochizuki, Institute of Human Genetics, Montpellier, France) was amplified with PrimeSTAR Max DNA polymerase (TaKaRa) and the following primers: forward, AAATAATAATACTAAACTTAAACAT and reverse, ACTAGTTGAGCGAACTGAATCGGTC. The resulting DNA contained the *MTT1* cadmium-inducible promoter [1], a *NEO5* drug-resistant marker, and the 5′ and 3′ UTRs of the *β-tubulin 1* genomic locus for homologous recombination, but no longer contained the EGFP cassette. The ORF of the mutated *APRT1* was amplified from genomic DNA of the homozygous homokaryons *mpr1-*

1/mpr1-1 (mpr1; 6mp^r) with PrimeSTAR Max DNA polymerase (TaKaRa) and the following primers: forward, aaataataatactaaacttaaacatATGGATTACTAAAATTAAGAAATAG and reverse, gaccgattcagttcgctcaactagtTTCAATATGAAGATTAATAACCTTG. The nucleotides in lowercase correspond to consensus sequences with the DNA ends of the backbone. The amplified fragment was integrated into the plasmid backbone with the NEBuilder HF DNA Assembly Kit (New England BioLabs). The resulting plasmid, pMTT1-D127-NEO5, was linearized with SacI and KpnI (New England BioLabs) before biolistic transformation into vegetative *T. thermophila* [2]. A control plasmid carrying the wild-type version of *APRT1* was also prepared. Ectopic expression was induced in cells carrying the constructs by adding 1 μg/ml $CdCl_2$ (Sigma-Aldrich) to induce *APRT1* overexpression from the *MTT1* promoter. (**A**) Map of the ectopic APRTase expression plasmid pMTT1-D127N-NEO5 with *NEO5,* a resistance cassette against the neomycin derivative paromomycin, cadmium-inducible *MTT1* promoter, mutated *APRT1*, and 5′ and 3′ UTRs of *BTU1*. The control plasmid carried the wild-type *APRT1* gene. (**B**) A schematic showing the *BTU1* locus (upper) and the plasmid vector (lower). (**C**) Overexpression of APRTase in the transformants was confirmed by RT-qPCR. RNAiso Blood (TaKaRa) was used to extract total RNA from approximately 1×10^6 cells that had been exposed to 1 μg/ml $CdCl_2$ for 24 h. RNA was treated with DNase (Promega, Tokyo, Japan) to eliminate contaminating genomic DNA and then purified with the GeneJET RNA Cleanup and Concentration Micro Kit (Thermo Fisher Scientific, Kanagawa, Japan). Purified RNA (1 μg) was reverse transcribed with ProtoScript II Reverse Transcriptase (New England BioLabs) and an oligo(dT15) primer. Quantitative real-time PCR was performed in triplicate with the KAPA SYBR Fast qPCR Kit (NIPPON Genetics, Tokyo, Japan) and the Chromo4 PCR system (Bio-Rad, Hercules, CA, USA). An *APRT1*-specific amplicon of approximately 680 bp was produced with the following primers: forward, AAATAAATTATAGATAGAAGTGAAAATGGATTAC and reverse, AAAAAAATTTAATTCCTACTATATCC. An *HSP70*-specific PCR product of approximately 500 bp was used as an internal control for normalization and was produced with the following primers: forward, ATCTCTTGGGTAAGTTCAACC and reverse, TTGAAGACTTCTTCCAAAG. Columns and attached bars correspond to the means of three identical measurements and their standard deviations, respectively. Asterisks show significant differences at $p < 0.01$ (as calculated by *t*-test). (**D**) Cell growth curves in the presence of 15 μg/ml 6mp and 1 μg/ml $CdCl_2$. Both transformants remained as sensitive to 6mp as 6mp^s control cells; irrespective of the mutation, they all died at 72 h. Only 6mp^r control cells grew, and they reached $>10^6$ cells/ml at 72 h. Points and attached bars correspond to mean measurements from three identical experiments and their standard deviations, respectively.

References

1. Shang, Y.; Song, X.; Bowen, J.; Corstanje, R.; Gao, Y.; Gaertig, J.; Gorovsky, M. A. A robust inducible-repressible promoter greatly facilitates gene knockouts, conditional expression, and overexpression of homologous and heterologous genes in *Tetrahymena thermophila*. *Proc. Natl. Acad. Sci.* **2002**, *99*, 3734–3739, doi:10.1073/pnas.052016199.
2. Cassidy-Hanley, D.; Bowen, J.; Lee, J. H.; Cole, E.; VerPlank, L. A.; Gaertig, J.; Gorovsky, M. A.; Bruns, P. J. Germline and somatic transformation of mating Tetrahymena thermophila by particle bombardment. *Genetics* **1997**, *146*, 135–147.
