# Supplementary material for: Resistance to 6-Methylpurine is Conferred by Defective Adenine Phosphoribosyltransferase in Tetrahymena
Source: Genes (Basel). 2018 Mar 23;9(4):179. doi: 10.3390/genes9040179 (PMC5924521; doi:10.3390/genes9040179)
Supplement: Supplementary file 1 [file genes-09-00179-s001.zip › Figure S7.pdf]

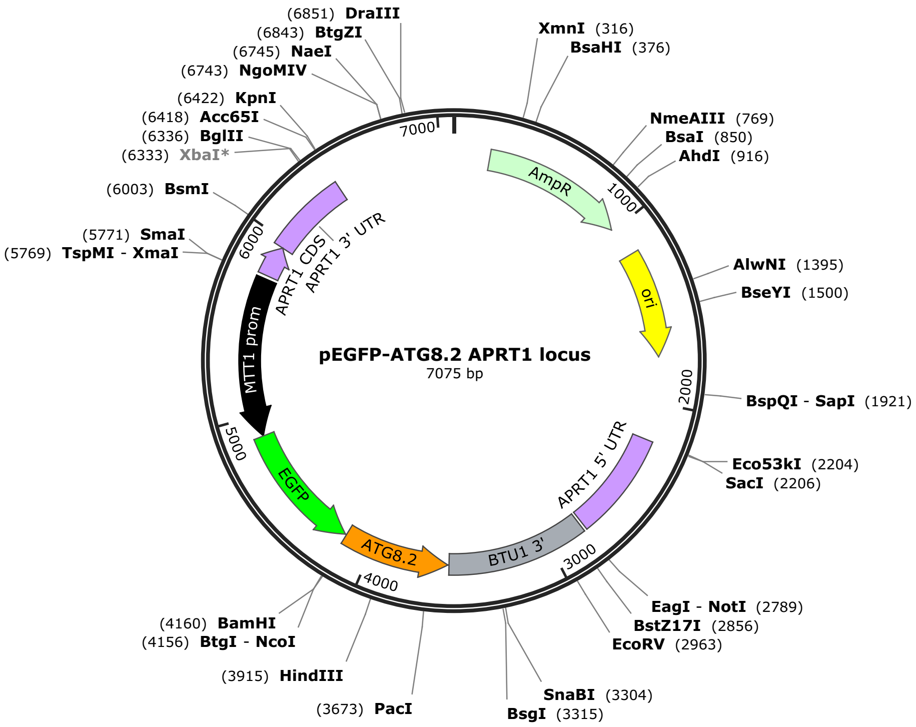

Figure S7. Map of trial plasmid pEGFP-ATG8.2 APRT1 locus. *NEO5* cassette of pΔAPRT1-NEO5 (Figure S2) was replaced by an EGFP-tagged ATG8.2–expressing cassette.

acgt: 5′ and 3′ UTRs of APRT1

ACGT: APRT1 CDS

3′ UTR of BTU1

ATG8.2 CDS

EGFP

MTT1 promoter

GTGGCACTTTTCGGGGAAATGTGCGCGGAACCCCTATTTGTTTATTTTTCTAAATACATTCAAATA
TGTATCCGCTCATGAGACAATAACCCTGATAAATGCTTCAATAATATTGAAAAAGGAAGAGTATGA
GTATTCAACATTTCCGTGTCGCCCTTATTCCCTTTTTTGCGGCATTTTGCCTTCCTGTTTTTGCTC
ACCCAGAAACGCTGGTGAAAGTAAAAGATGCTGAAGATCAGTTGGGTGCACGAGTGGGTTACATCG
AACTGGATCTCAACAGCGGTAAGATCCTTGAGAGTTTTCGCCCCGAAGAACGTTTTCCAATGATGA
GCACTTTTAAAGTTCTGCTATGTGGCGCGGTATTATCCCGTATTGACGCCGGGCAAGAGCAACTCG
GTCGCCGCATACACTATTCTCAGAATGACTTGGTTGAGTACTCACCAGTCACAGAAAAGCATCTTA
CGGATGGCATGACAGTAAGAGAATTATGCAGTGCTGCCATAACCATGAGTGATAACACTGCGGCCA
ACTTACTTCTGACAACGATCGGAGGACCGAAGGAGCTAACCGCTTTTTTGCACAACATGGGGGATC
ATGTAACTCGCCTTGATCGTTGGGAACCGGAGCTGAATGAAGCCATACCAAACGACGAGCGTGACA
CCACGATGCCTGTAGCAATGGCAACAACGTTGCGCAAACTATTAACTGGCGAACTACTTACTCTAG

CTTCCCGGCAACAATTAATAGACTGGATGGAGGCGGATAAAGTTGCAGGACCACTTCTGCGCTCGG
CCCTTCCGGCTGGCTGGTTTATTGCTGATAAATCTGGAGCCGGTGAGCGTGGGTCTCGCGGTATCA
TTGCAGCACTGGGGCCAGATGGTAAGCCCTCCCGTATCGTAGTTATCTACACGACGGGGAGTCAGG
CAACTATGGATGAACGAAATAGACAGATCGCTGAGATAGGTGCCTCACTGATTAAGCATTGGTAAC
TGTCAGACCAAGTTTACTCATATATACTTTAGATTGATTTAAAACTTCATTTTTAATTTAAAAGGA
TCTAGGTGAAGATCCTTTTTGATAATCTCATGACCAAAATCCCTTAACGTGAGTTTTCGTTCCACT
GAGCGTCAGACCCCGTAGAAAAGATCAAAGGATCTTCTTGAGATCCTTTTTTTCTGCGCGTAATCT
GCTGCTTGCAAACAAAAAAACCACCGCTACCAGCGGTGGTTTGTTTGCCGGATCAAGAGCTACCAA
CTCTTTTTCCGAAGGTAACTGGCTTCAGCAGAGCGCAGATACCAAATACTGTCCTTCTAGTGTAGC
CGTAGTTAGGCCACCACTTCAAGAACTCTGTAGCACCGCCTACATACCTCGCTCTGCTAATCCTGT
TACCAGTGGCTGCTGCCAGTGGCGATAAGTCGTGTCTTACCGGGTTGGACTCAAGACGATAGTTAC
CGGATAAGGCGCAGCGGTCGGGCTGAACGGGGGGTTCGTGCACACAGCCCAGCTTGGAGCGAACGA
CCTACACCGAACTGAGATACCTACAGCGTGAGCTATGAGAAAGCGCCACGCTTCCCGAAGGGAGAA
AGGCGGACAGGTATCCGGTAAGCGGCAGGGTCGGAACAGGAGAGCGCACGAGGGAGCTTCCAGGGG
GAAACGCCTGGTATCTTTATAGTCCTGTCGGGTTTCGCCACCTCTGACTTGAGCGTCGATTTTTGT
GATGCTCGTCAGGGGGGCGGAGCCTATGGAAAAACGCCAGCAACGCGGCCTTTTTACGGTTCCTGG
CCTTTTGCTGGCCTTTTGCTCACATGTTCTTTCCTGCGTTATCCCCTGATTCTGTGGATAACCGTA
TTACCGCCTTTGAGTGAGCTGATACCGCTCGCCGCAGCCGAACGACCGAGCGCAGCGAGTCAGTGA
GCGAGGAAGCGGAAGAGCGCCCAATACGCAAACCGCCTCTCCCCGCGCGTTGGCCGATTCATTAAT
GCAGCTGGCACGACAGGTTTCCCGACTGGAAAGCGGGCAGTGAGCGCAACGCAATTAATGTGAGTT
AGCTCACTCATTAGGCACCCCAGGCTTTACACTTTATGCTTCCGGCTCGTATGTTGTGTGGAATTG
TGAGCGGATAACAATTTCACACAGGAAACAGCTATGACCATGATTACGCCAAGCGCGCAATTAACC
CTCACTAAAGGGAACAAAAGCTGGAGCTCgagctgaatgaatgaatgaatgtatttatgaat
gaattaatgcaataaataataaaataatactttttataatatttagtttaaaattatatgtctact
atatataaatattctttaaactccatcttaaagacaccaaaataccttagctatttattaggtttt
ataacaaaaatcatttgatttttagttaagcaaacaagtaagcaatggttttaaatacaataaat
aattttatttatcttaaaatcaaattttattgaagatttttattcaaataaatttctttattat
tcgttttgagtattttatcatcctcgcttactgattttgttcaaaatatccttttattgatattaa
ttgcttaaattaattagagttaagctcaatattttgagtgaaatttgaaaaattctgaaattattt
tcatttggaatttgcttttcaagatattctattttgtaataaaaataaaaatattaagacaattaa
ttagaaaaatagataaatatcactctattttcaggatttgctttactttctttttgattgatttgt
ggggtaataaaattGGCGGCCGCGTCGAGAAGATGTGGCTATTGATGGGCATAAAGATTTTATTTA
AATAAAGGGGTTTTTGTATACAAGGGAAAATAAATTTTGTTTTTGAAAAATAAAAAGATATTTCTT
TAATTTAAAAAAAAAAATTGGCAAGTAAGTAAAAGAGAGGAAAACAAGTTAAAAAGAGATATCAACT
TATTTATTCATTCATAGATATTTAATTTAAAAAAGAAGTTTATCAATCTTGAAGTAATAACAAATT
TATTTATAGTACTGAAAAGCAATTAAAAAACTATAAAAACAACTTGGGTTTGTTTTGTTGCTTTAA
TATTATGCTGACTGACTGATTGATTGAAAATATTTGTTTTAAATAATTCAAAATAAAAAAGGATTT
TGTTTATTTTTTTATTGTATTTATTTTTGTATGCTATTTATAAAATAATTTTGTAATAATAAGAAA
GGAAACATGTAGAAGTAAGAATTTTTTTGGTATGTTTTTGTTGTGAAAAATGCGGGTGAGTGCAGA
ATACGTATTTATATAAGAACTATTTTGTAAAAAGAGATATATTATTTTGTTAATATATATCCATTA
TTTTTAAAAAGTTATTTGTTGATTTAGAGAGTTGTATATATGTGAGAAAAAAGATGTTTATAGATA
TTAATGTTATGTGAATGAAGTTAATTGGGTATTTCTTTCTCTACTATTACAACAACAGTAAGATGC
TTAAGTAAAAATAATAAAGTTTATTATGTTGATTGGTTGGTTTAGCTGACCGATTCAGTTCGCTCA
ACTAGTAATTGATCCAAATACTTCTTGATCAGAGTATGTGATATATAAGAAACCATCATCCTTATC

CTTTGATTCTTGGTAAATTTCGCTCATTGTTCTATCTATTAATTAATTATAAGATTAAAATTAATG
AAAATTAATTCAAAGTTTTGATTGCTTAATTTTAGAATTCATTTTATTTAGATAGATTGTTTTTGA
ATGAATTTCTACATTCAACTTAAATAATTGATTAAAAAGGAAAAATAATATTTACTTATTTTCTCA
ATCTTTTTATTGCCAACAAAGAAGAACAAAGCATCAGCTTCATTTGTAATATCTAATTTCTTACGA
ACTGTTTATTGGAATTCGTGAAGCTTAACATCCTTTGATACCAAGAACCTTTTTGTAAATGAAATA
AATTAATCAAAGTTTTTAAAATCATTATTAAATTTGTTTACTTTTGCTTTTCCAATACTCTAACTG
TTTTTGCCTTCTTGTTAATTTCAACAATGACGGGAATCATGTTTGGGTATTTAGATTTCTACTTTT
CTAATTCTTTCTATCTTTCTGCTAGAGAGAATTTTTCCTTGAAGGGTTTATAATTTTGAGCGTCCA
TGGATCCCTTATATAATTCATCCATACCAAGAGTAATACCAGCAGCAGTAACAAATTCTAAAAGAA
CCATATGATCTCTTTTTTCATTAGGATCTTTAGATAAAGCTGATTGAGTAGATAAGTAGTGATTAT
CAGGTAAAAGAACAGGACCATCACCAATAGGAGTATTTTGTTGATAATGATCAGCTAATTGAACTG
AACCATCTTCAATATTATGTCTAATTTTAAAATTAACTTTAATACCATTCTTTTGCTTATCAGCCA
TAATGTAAACATTATGAGAATTGTAATTATATTCAAGTTTATGACCAAGAATATTACCATCTTCCT
TGAAATCAATACCTTTAAGTTCAATTCTATTAACTAAAGTATCACCTTCAAATTTAACTTCAGCTC
TAGTTTTATAATTACCATCATCTTTAAAGAAAATAGTTCTTTCTTGAACATAACCTTCAGGCATAG
CAGACTTAAAGAAATCATGTTGCTTCATGTGATCAGGGTATCTTGAAAAGCATTGAACACCATAAG
TAAGAGTAGTAACTAAAGTAGGCCAAGGAACAGGTAACTTACCAGTAGTACAAATAAATTTAAGAG
TTAACTTACCATAAGTAGCATCACCTTCACCTTCACCTGAAACAGAAAATTTGTGACCATTAACAT
CACCATCTAATTCAACTAAAATAGGAACAACACCAGTGAAAAGTTCTTCACCCTTAGAAACCATAT
GTTTAAGTTTAGTATTATTATTTATTTTATTAGAGCTTTATTAAATTTTTTTAATTTTTTTAAATT
ATATAAAGAATAAAAAAGACGAATATATATATATACACTATTTACATTATTTTATATGGATCATTG
CATAAATCGTGAATCACGTAGCTAAGAATTATATCAGAAATATAAAAAATTACTTTATATTCAAGA
GAGATTCAAGAATCACATCTATATTTTAGAATAGAAGAATTTTGAAAATTAGTTAGGTTGACTCAT
GATTTAAATCATGAGTCAATCAATTTATATTTTTTATCAGAAATAAAAAGATTTACAAATAATTCA
TGACACAAAATTCAAGAATCACAACTTAATATTAAAATATAATAGAAACGGATAATTGAAAATAAA
AAATAAATGATAGCCTAAATAATGAGTAATATTTTGAAAATTAATGATTCACATATTATAATTGAT
GAATGAGCTATGTTTTGAGCAGCTTATATATTTAATAAATAAAATAATTGATATTTATCTATTTTA
TATTTCATGTTTTCTTTAAAAAACATGTCATCTTTTTTATCAATATATTTGAAATTTAAAGAAAAT
AATTGAATAAACGATACAATATATATTTTAAGATATATATAAAAAAGTTTTGCTTTCAAGATATTAAAAA
TAGTGATATAAAAAAATAAGTACTCTATTATGTTTTTTCTTATTCAGTATTATACCTTTAATCATTA
TTATCTTTTTATTTATTTTTAGTTAGTTATTTTTTATTTTTATGAATATTTAAAGAGCTAAAAAAA
ATTTAAAAATGTGTATTTAAATTAAAGGAGTTATTCAAAACCCTTATTATTTTTTATTTTTAAATA
TTTTTTAGAAATAAATTGTCTGATCCCCCGGGGGATCAGCTCGAGGGATCTTAAGGGCGAAGAAAG
ATTATCTAAATACAAGGTTATTAATCTTCATATTGAATGAataagtgaggtagtgataaaaataaa
ataaattgaatgaacaagttagtttgtttttgtatgaatgtgttttatatttatggatatagtagg
aattaaatttttttgatttagtaatttagtatgtacttcttaaagtttaaataataagatttgcat
tcaatttttgtaaattaagtagttttatgtttttttaactacataagtatttatcattgaatgtaat
tagttaactaaaatttttttatttcaacatttttaaagaaagatttttactctttttgttgttattt
atttattgttaaaaaggcttattaaaatagattgaattataaacaaaaagaataattaaaaaaacag
aatgaataaatgatctttctatatatataaatatttttaattttcattaaattttataataaaaaa
tttatgatattattaattgactagttagctggtatcgtatatatatcctgcttttaaggaagtcta
gatctattaaattttaatttaccttagcatgaggttacttaaatatttatttgataggatgtcaaa
ggtcataaataaacgGGTACCCAATTCGCCCTATAGTGAGTCGTATTACGCGCGCTCACTGGCCGT
CGTTTTACAACGTCGTGACTGGGAAAACCCTGGCGTTACCCAACTTAATCGCCTTGCAGCACATCC

CCCTTTCGCCAGCTGGCGTAATAGCGAAGAGGCCCGCACCGATCGCCCTTCCCAACAGTTGCGCAG
CCTGAATGGCGAATGGGACGCGCCCTGTAGCGGCGCATTAAGCGCGGCGGGTGTGGTGGTTACGCG
CAGCGTGACCGCTACACTTGCCAGCGCCCTAGCGCCCGCTCCTTTCGCTTTCTTCCCTTCCTTTCT
CGCCACGTTCGCCGGCTTTCCCCGTCAAGCTCTAAATCGGGGGCTCCCTTTAGGGTTCCGATTTAG
TGCTTTACGGCACCTCGACCCCAAAAAACTTGATTAGGGTGATGGTTCACGTAGTGGGCCATCGCC
CTGATAGACGGTTTTTCGCCCTTTGACGTTGGAGTCCACGTTCTTTAATAGTGGACTCTTGTTCCA
AACTGGAACAACACTCAACCCTATCTCGGTCTATTCTTTTGATTTATAAGGGATTTTGCCGATTTC
GGCCTATTGGTTAAAAAATGAGCTGATTTAACAAAAATTTAACGCGAATTTTAACAAAATATTAAC
GCTTACAATTTAG
