# Supplementary material for: Resistance to 6-Methylpurine is Conferred by Defective Adenine Phosphoribosyltransferase in Tetrahymena
Source: Genes (Basel). 2018 Mar 23;9(4):179. doi: 10.3390/genes9040179 (PMC5924521; doi:10.3390/genes9040179)
Supplement: Supplementary file 1 [file genes-09-00179-s001.zip › Figure S6.pdf]

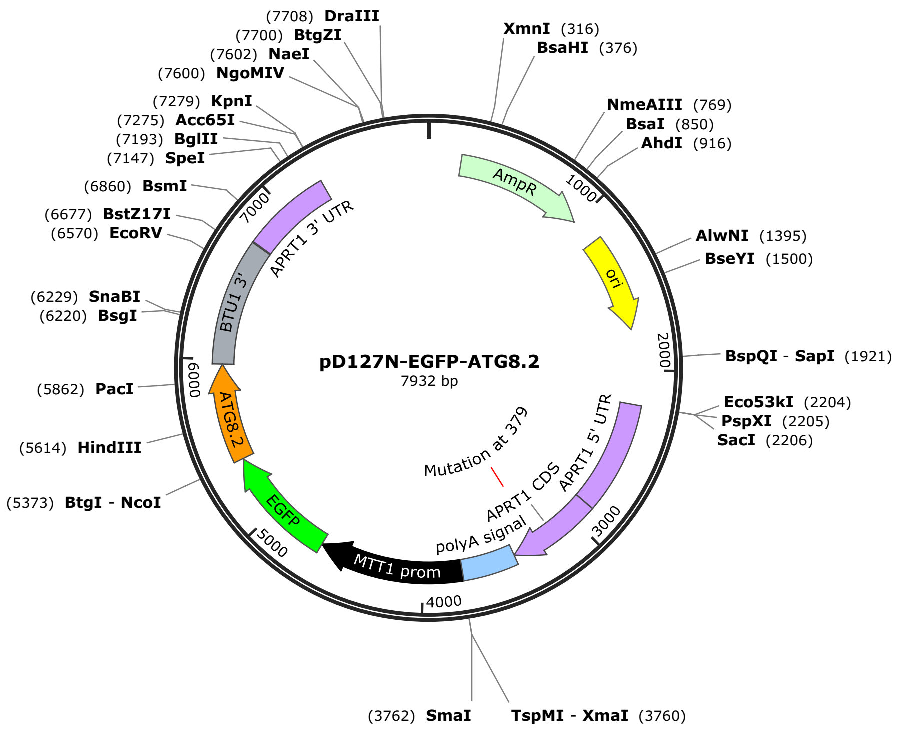

Figure S6. Map of trial plasmid pD127N-EGFP-ATG8.2. *PAC* cassette of pD127N-FZZ-PAC (Figure S1) was replaced by an EGFP-tagged ATG8.2–expressing cassette.

acgt: 5′ and 3′ UTRs of APRT1

ACGT: APRT1 CDS

A: Mutation at 379

polyA signal

MTT1 promoter

EGFP

ATG8.2 CDS

3′ UTR of BTU1

GTGGCACTTTTCGGGGAAATGTGCGCGGAACCCCTATTTGTTTATTTTTCTAAATACATTCAAATA
TGTATCCGCTCATGAGACAATAACCCTGATAAATGCTTCAATAATATTGAAAAAGGAAGAGTATGA
GTATTCAACATTTCCGTGTCGCCCTTATTCCCTTTTTTGCGGCATTTTGCCTTCCTGTTTTTGCTC
ACCCAGAAACGCTGGTGAAAGTAAAAGATGCTGAAGATCAGTTGGGTGCACGAGTGGGTTACATCG
AACTGGATCTCAACAGCGGTAAGATCCTTGAGAGTTTTCGCCCCGAAGAACGTTTTCCAATGATGA
GCACTTTTAAAGTTCTGCTATGTGGCGCGGTATTATCCCGTATTGACGCCGGGCAAGAGCAACTCG
GTCGCCGCATACACTATTCTCAGAATGACTTGGTTGAGTACTCACCAGTCACAGAAAAGCATCTTA
CGGATGGCATGACAGTAAGAGAATTATGCAGTGCTGCCATAACCATGAGTGATAACACTGCGGCCA
ACTTACTTCTGACAACGATCGGAGGACCGAAGGAGCTAACCGCTTTTTTGCACAACATGGGGGATC

ATGTAACTCGCCTTGATCGTTGGGAACCGGAGCTGAATGAAGCCATACCAAACGACGAGCGTGACA
CCACGATGCCTGTAGCAATGGCAACAACGTTGCGCAAACTATTAACTGGCGAACTACTTACTCTAG
CTTCCCGGCAACAATTAATAGACTGGATGGAGGCGGATAAAGTTGCAGGACCACTTCTGCGCTCGG
CCCTTCCGGCTGGCTGGTTTATTGCTGATAAATCTGGAGCCGGTGAGCGTGGGTCTCGCGGTATCA
TTGCAGCACTGGGGCCAGATGGTAAGCCCTCCCGTATCGTAGTTATCTACACGACGGGGAGTCAGG
CAACTATGGATGAACGAAATAGACAGATCGCTGAGATAGGTGCCTCACTGATTAAGCATTGGTAAC
TGTCAGACCAAGTTTACTCATATATACTTTAGATTGATTTAAAACTTCATTTTTAATTTAAAAGGA
TCTAGGTGAAGATCCTTTTTGATAATCTCATGACCAAAATCCCTTAACGTGAGTTTTCGTTCCACT
GAGCGTCAGACCCCGTAGAAAAGATCAAAGGATCTTCTTGAGATCCTTTTTTTCTGCGCGTAATCT
GCTGCTTGCAAACAAAAAAACCACCGCTACCAGCGGTGGTTTGTTTGCCGGATCAAGAGCTACCAA
CTCTTTTTCCGAAGGTAACTGGCTTCAGCAGAGCGCAGATACCAAATACTGTCCTTCTAGTGTAGC
CGTAGTTAGGCCACCACTTCAAGAACTCTGTAGCACCGCCTACATACCTCGCTCTGCTAATCCTGT
TACCAGTGGCTGCTGCCAGTGGCGATAAGTCGTGTCTTACCGGGTTGGACTCAAGACGATAGTTAC
CGGATAAGGCGCAGCGGTCGGGCTGAACGGGGGGTTCGTGCACACAGCCCAGCTTGGAGCGAACGA
CCTACACCGAACTGAGATACCTACAGCGTGAGCTATGAGAAAGCGCCACGCTTCCCGAAGGGAGAA
AGGCGGACAGGTATCCGGTAAGCGGCAGGGTCGGAACAGGAGAGCGCACGAGGGAGCTTCCAGGGG
GAAACGCCTGGTATCTTTATAGTCCTGTCGGGTTTCGCCACCTCTGACTTGAGCGTCGATTTTTGT
GATGCTCGTCAGGGGGGCGGAGCCTATGGAAAAACGCCAGCAACGCGGCCTTTTTACGGTTCCTGG
CCTTTTGCTGGCCTTTTGCTCACATGTTCTTTCCTGCGTTATCCCCTGATTCTGTGGATAACCGTA
TTACCGCCTTTGAGTGAGCTGATACCGCTCGCCGCAGCCGAACGACCGAGCGCAGCGAGTCAGTGA
GCGAGGAAGCGGAAGAGCGCCCAATACGCAAACCGCCTCTCCCCGCGCGTTGGCCGATTCATTAAT
GCAGCTGGCACGACAGGTTTCCCGACTGGAAAGCGGGCAGTGAGCGCAACGCAATTAATGTGAGTT
AGCTCACTCATTAGGCACCCCAGGCTTTACACTTTATGCTTCCGGCTCGTATGTTGTGTGGAATTG
TGAGCGGATAACAATTTCACACAGGAAACAGCTATGACCATGATTACGCCAAGCGCGCAATTAACC
CTCACTAAAGGGAACAAAAGCTGGAGCTCgagctgaatgaatgaatgaatgaatgtatttatgaat
gaattaatgcaataaataataaaataatactttttataatatttagtttaaaattatatgtctact
atatataaatattctttaaactccatcttaaagacaccaaaataccttagctatttattaggtttt
ataacaaaaatcatttgatttttttagttaagcaaacaagtaagcaatggttttaaatacaataaat
aattttatttatcttaaaatcaaattttattgaagatttttttattcaaataaatttttctttattat
tcgttttgagtattttatcatcctcgcttactgattttgttcaaaatatccttttattgatattaa
ttgcttaaattaattagagttaagctcaatattttgagtgaaatttgaaaaattctgaaattattt
tcatttggaatttgcttttcaagatattctattttgtaataaaaataaaaatattaagacaattaa
ttagaaaaatagataaatatcactctattttcaggatttgctttactttctttttgattgatttgt
ggggtaataaaattaggataactaagtctgcttaattttaataaaataaataattattgatttaca
aagatattctcttaaaaaaataaattatagatagaagtgaaaATGGATTACTAAAATTAAGAAATA
GGTAAAGTTATTAGCGAAAACTTCAGAAATATTCCTGATTTCCCTTAACCTGGTATTATCTTTAAA
GATATTGCTCCTCTCCTCTCTAATCCTGCCATTTTTAAGCGTATTACTGATGCTTTTGTAATTGCT
TTGAAGGACATTGATTTTGATAAAATTGTTATCTTAGAATCTAGAGGTTTTATTTTTGGAACCGCT
ATTGGTTTACAAATGGGAAAGGGATTAATTTTAATCAGAAAGAAAGGCAAACTACCTGGTAAGGTT
GAATAATTCTCATTCGACTTAGAATACGGTAAGGATGTTTTTGAAATTCAAAGTGATGCTTTAGCT
AAAGGTGAAAAAGTTGTTATTGTTAATGATATTCTAGCTACTGGTGGAACTGCTGATGCAGCTTGC
AGATTAGTTGAAAAGGTAGGAGGAGTAGTATAAGCTGTTATGTTCCTTTACCAAATTAAGGATCTT
AAGGGCGAAGAAAGATTATCTAAATACAAGGTTATTAATCTTCATATTGAATGAGATCCTTAAATT
AAAAATTCAATATATATTTACAAACTTTCATATAAAATAAATATATTATATAAAATTAATTTTAG

TGTATTATATTAACATTAAAGCACCAAAAAAACGTGTTAATATACTACTATAAAATATAATTTATT
CCAAATTGACTAAAATCATTATTTTACAACTCATTTGTATATATATTTTATGTCAATTATTTTTTT
TAACTTTCTAAAAAAAAAAATTCCTCTTCACATACATGTTAGCTCTTAAAAATTTGTCTGCAAATC
CAATAATAATATTTTTTTTTGCCATTAAATTTTCAAATTTTTACTGGAAAAATGCAGCCCGGGGGA
TCAGACAATTTATTTCTAAAAAATATTTAAAAATAAAAAATAATAAGGGTTTTGAATAACTCCTTT
AATTTAAATACACATTTTTAAATTTTTTTTAGCTCTTTAAATATTCATAAAAATAAAAAATAACTA
ACTAAAAATAAATAAAAAGATAATAATGATTAAAGGTATAATACTGAATAAGAAAAAACATAATAG
AGTACTTATTTTTTATATCACTATTTTTAATATCTTGAAAGCAAAACTTTTTTATATATCTTAAAA
TATATTGTATCGTTTATTCAATTATTTTCTTTAAATTTCAAATATATTGATAAAAAAGATGACATG
TTTTTTAAAGAAAACATGAAATATAAAATAGATAAATATCAATTATTTTATTTATTAAATATATAA
GCTGCTCAAAACATAGCTCATTCATCAATTATAATATGTGAATCATTAATTTTCAAAATATTACTC
ATTATTTAGGCTATCATTTATTTTTTATTTTCAATTATCCGTTTCTATTATATTTTAATATTAAGT
TGTGATTCTTGAATTTTGTGTCATGAATTATTTGTAAATCTTTTTATTTCTGATAAAAAATATAAA
TTGATTGACTCATGATTTAAATCATGAGTCAACCTAACTAATTTTCAAAATTCTTCTATTCTAAAA
TATAGATGTGATTCTTGAATCTCTCTTGAATATAAAGTAATTTTTTATATTTCTGATATAATTCTT
AGCTACGTGATTCACGATTTATGCAATGATCCATATAAAATAATGTAAATAGTGTATATATATATA
TTCGTCTTTTTTATTCTTTATATAATTTAAAAAAATTAAAAAAATTTAATAAAGCTCTAATAAAAT
AAATAATAATACTAAACTTAAACATATGGTTTCTAAGGGTGAAGAACTTTTCACTGGTGTTGTTCC
TATTTTAGTTGAATTAGATGGTGATGTTAATGGTCACAAATTTTCTGTTTCAGGTGAAGGTGAAGG
TGATGCTACTTATGGTAAGTTAACTCTTAAATTTATTTGTACTACTGGTAAGTTACCTGTTCCTTG
GCCTACTTTAGTTACTACTCTTACTTATGGTGTTCAATGCTTTTCAAGATACCCTGATCACATGAA
GCAACATGATTTCTTTAAGTCTGCTATGCCTGAAGGTTATGTTCAAGAAAGAACTATTTTCTTTAA
AGATGATGGTAATTATAAAACTAGAGCTGAAGTTAAATTTGAAGGTGATACTTTAGTTAATAGAAT
TGAACTTAAAGGTATTGATTTCAAGGAAGATGGTAATATTCTTGGTCATAAACTTGAATATAATTA
CAATTCTCATAATGTTTACATTATGGCTGATAAGCAAAAGAATGGTATTAAAGTTAATTTTAAAAT
TAGACATAATATTGAAGATGGTTCAGTTCAATTAGCTGATCATTATCAACAAAATACTCCTATTGG
TGATGGTCCTGTTCTTTTACCTGATAATCACTACTTATCTACTCAATCAGCTTTATCTAAAGATCC
TAATGAAAAAAGAGATCATATGGTTCTTTTAGAATTTGTTACTGCTGCTGGTATTACTCTTGGTAT
GGATGAATTATATAAGGGATCCATGGACGCTCAAAATTATAAACCCTTCAAGGAAAAATTCTCTCT
AGCAGAAAGATAGAAAGAATTAGAAAAGTAGAAATCTAAATACCCAAACATGATTCCCGTCATTGT
TGAAATTAACAAGAAGGCAAAAACAGTTAGAGTATTGGAAAAGCAAAAGTAAACAAATTTAATAAT
GATTTTAAAAACTTTGATTAATTTATTTCATTTACAAAAAGGTTCTTGGTATCAAAGGATGTTAAG
CTTCACGAATTCCAATAAACAGTTCGTAAGAAATTAGATATTACAAATGAAGCTGATGCTTTGTTC
TTCTTTGTTGGCAATAAAAAGATTGAGAAAATAAGTAAATATTATTTTTCCTTTTTAATCAATTAT
TTAAGTTGAATGTAGAAATTCATTCAAAAACAATCTATCTAAATAAAATGAATTCTAAAATTAAGC
AATCAAAACTTTGAATTAATTTTCATTAATTTTAATCTTATAATTAATTAATAGATAGAACAATGA
GCGAAATTTACCAAGAATCAAAGGATAAGGATGATGGTTTCTTATATATCACATACTCTGATCAAG
AAGTATTTGGATCAATTACTGATTGAGCGAACTGAATCGGTCAGCTAAACCAACCAATCAACATAA
TAAACTTTATTATTTTTACTTAAGCATCTTACTGTTGTTGTAATAGTAGAGAAAGAAATACCCAAT
TAACTTCATTCACATAACATTAATATCTATAAACATCTTTTTTCTCACATATATACAACTCTCTAA
ATCAACAAATAACTTTTTAAAAATAATGGATATATATTAACAAAATAATATATCTCTTTTTACAAA
ATAGTTCTTATATAAATACGTATTCTGCACTCACCCGCATTTTTCACAACAAAAACATACCAAAAA
AATTCTTACTTCTACATGTTTCCTTTCTTATTATTACAAAATTATTTTATAAATAGCATACAAAAA
TAAATACAATAAAAAAATAAACAAAATCCTTTTTTATTTTGAATTATTTAAAACAAATATTTTCAA

TCAATCAGTCAGTCAGCATAATATTAAAGCAACAAAACAAACCCAAGTTGTTTTTATAGTTTTTTA
ATTGCTTTTCAGTACTATAAATAAATTTGTTATTACTTCAAGATTGATAAACTTCTTTTTTAAATT
AAATATCTATGAATGAATAAATAAGTTGATATCTCTTTTTAACTTGTTTTCCTCTCTTTTACTTAC
TTGCCAATTTTTTTTTTAAATTAAAGAAATATCTTTTTATTTTTCAAAAACAAAATTTATTTTCCC
TTGTATACAAAAACCCCTTTATTTAAATAAAATCTTTATGCCCATCAATAGCCACATCTTCTCGAG
gaatgaacaagttagtttgtttttgtatgaatgtgttttatatttatggatatagtaggaattaaa
tttttttgatttagtaatttagtatgtacttcttaaagtttaaataataagatttgcattcaattt
ttgtaaattaagtagttttatgtttttttaactacataagtatttatcattgaatgtaattagttaa
ctaaaatttttttatttcaacatttttaaagaaagatttttactctttttgttgttatttatttatt
gttaaaaaggcttattaaaatagattgaattataaacaaaaagaataattaaaaaacagaatgaat
aaatgatctttctatatatataaatatttttaattttcattaaattttataataaaaaatttatga
tattattaattgactagttagctggtatcgtatatatatcctgcttttaaggaagtctagatctat
taaattttaatttaccttagcatgaggttacttaaatatttatttgataggatgtcaaaggtcata
aataaacgGGTACCCAATTCGCCCTATAGTGAGTCGTATTACGCGCGCTCACTGGCCGTCGTTTTA
CAACGTCGTGACTGGGAAAACCCTGGCGTTACCCAACTTAATCGCCTTGCAGCACATCCCCCTTTC
GCCAGCTGGCGTAATAGCGAAGAGGCCCGCACCGATCGCCCTTCCCAACAGTTGCGCAGCCTGAAT
GGCGAATGGGACGCGCCCTGTAGCGGCGCATTAAGCGCGGCGGGTGTGGTGGTTACGCGCAGCGTG
ACCGCTACACTTGCCAGCGCCCTAGCGCCCGCTCCTTTCGCTTTCTTCCCTTCCTTTCTCGCCACG
TTCGCCGGCTTTCCCCGTCAAGCTCTAAATCGGGGGCTCCCTTTAGGGTTCCGATTTAGTGCTTTA
CGGCACCTCGACCCCAAAAAACTTGATTAGGGTGATGGTTCACGTAGTGGGCCATCGCCCTGATAG
ACGGTTTTTCGCCCTTTGACGTTGGAGTCCACGTTCTTTAATAGTGGACTCTTGTTCCAAACTGGA
ACAACACTCAACCCTATCTCGGTCTATTCTTTTGATTTATAAGGGATTTTGCCGATTTCGGCCTAT
TGGTTAAAAAATGAGCTGATTTAACAAAAATTTAACGCGAATTTTAACAAAATATTAACGCTTACA
ATTTAG
