# Supplementary material for: Resistance to 6-Methylpurine is Conferred by Defective Adenine Phosphoribosyltransferase in Tetrahymena
Source: Genes (Basel). 2018 Mar 23;9(4):179. doi: 10.3390/genes9040179 (PMC5924521; doi:10.3390/genes9040179)
Supplement: Supplementary file 1 [file genes-09-00179-s001.zip › Figure S4.pdf]

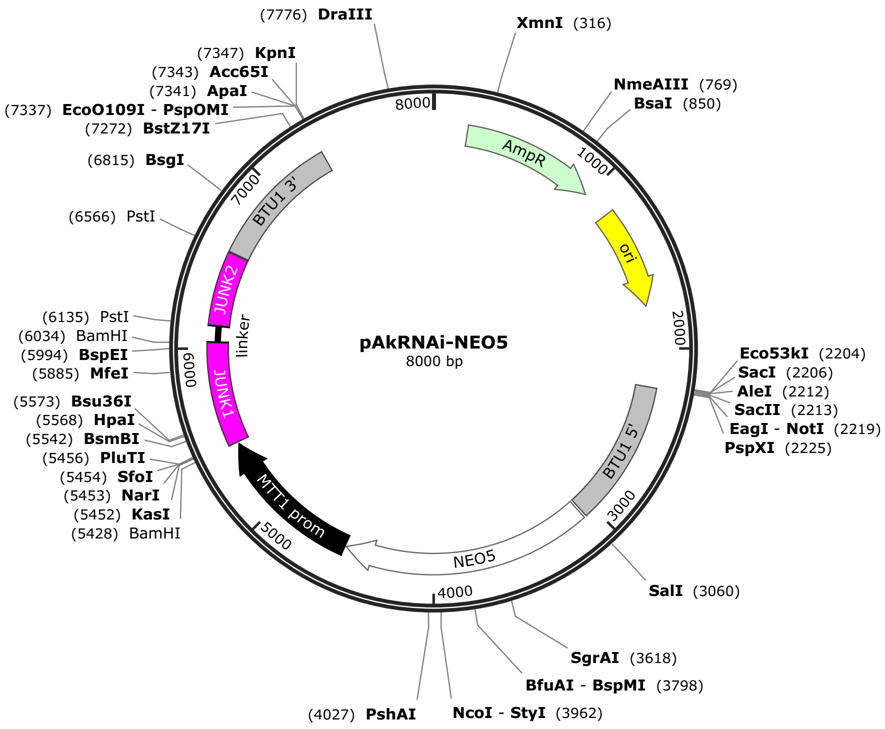

Figure S4. Map of the RNAi backbone plasmid pAkRNAi-NEO5, with paromomycin resistance cassette (*NEO5*), *MTT1* promoter, junk DNA sequences separated by a linker sequence, and 5′ and 3′ UTRs of *BTU1*. Target sequences to create the hairpin construct were cloned into BamHI–BamHI sites and PstI–PstI I sites. The entire sequence of the plasmid is depicted at the bottom of the figure.

5′ and 3′ UTRs of BTU1

NEO5 cassette

MTT1 promoter

Junk sequences

Linker

GTGGCACTTTTCGGGGAAATGTGCGCGGAACCCCTATTTGTTTATTTTTCTAAATACATTCAAATA
TGTATCCGCTCATGAGACAATAACCCTGATAAATGCTTCAATAATATTGAAAAAGGAAGAGTATGA
GTATTCAACATTTCCGTGTCGCCCTTATTCCCTTTTTTGCGGCATTTTGCCTTCCTGTTTTTGCTC
ACCCAGAAACGCTGGTGAAAGTAAAAGATGCTGAAGATCAGTTGGGTGCACGAGTGGGTTACATCG
AACTGGATCTCAACAGCGGTAAGATCCTTGAGAGTTTTCGCCCCGAAGAACGTTTTCCAATGATGA
GCACTTTTAAAGTTCTGCTATGTGGCGCGGTATTATCCCGTATTGACGCCGGGCAAGAGCAACTCG
GTCGCCGCATACACTATTCTCAGAATGACTTGGTTGAGTACTCACCAGTCACAGAAAAGCATCTTA
CGGATGGCATGACAGTAAGAGAATTATGCAGTGCTGCCATAACCATGAGTGATAACACTGCGGCCA
ACTTACTTCTGACAACGATCGGAGGACCGAAGGAGCTAACCGCTTTTTTGCACAACATGGGGGATC

ATGTAACTCGCCTTGATCGTTGGGAACCGGAGCTGAATGAAGCCATACCAAACGACGAGCGTGACA
CCACGATGCCTGTAGCAATGGCAACAACGTTGCGCAAACTATTAACTGGCGAACTACTTACTCTAG
CTTCCCGGCAACAATTAATAGACTGGATGGAGGCGGATAAAGTTGCAGGACCACTTCTGCGCTCGG
CCCTTCCGGCTGGCTGGTTTATTGCTGATAAATCTGGAGCCGGTGAGCGTGGGTCTCGCGGTATCA
TTGCAGCACTGGGGCCAGATGGTAAGCCCTCCCGTATCGTAGTTATCTACACGACGGGGAGTCAGG
CAACTATGGATGAACGAAATAGACAGATCGCTGAGATAGGTGCCTCACTGATTAAGCATTGGTAAC
TGTCAGACCAAGTTTACTCATATATACTTTAGATTGATTTAAAACTTCATTTTTAATTTAAAAGGA
TCTAGGTGAAGATCCTTTTTGATAATCTCATGACCAAAATCCCTTAACGTGAGTTTTCGTTCCACT
GAGCGTCAGACCCCGTAGAAAAGATCAAAGGATCTTCTTGAGATCCTTTTTTTCTGCGCGTAATCT
GCTGCTTGCAAACAAAAAAACCACCGCTACCAGCGGTGGTTTGTTTGCCGGATCAAGAGCTACCAA
CTCTTTTTCCGAAGGTAACTGGCTTCAGCAGAGCGCAGATACCAAATACTGTCCTTCTAGTGTAGC
CGTAGTTAGGCCACCACTTCAAGAACTCTGTAGCACCGCCTACATACCTCGCTCTGCTAATCCTGT
TACCAGTGGCTGCTGCCAGTGGCGATAAGTCGTGTCTTACCGGGTTGGACTCAAGACGATAGTTAC
CGGATAAGGCGCAGCGGTCGGGCTGAACGGGGGGTTCGTGCACACAGCCCAGCTTGGAGCGAACGA
CCTACACCGAACTGAGATACCTACAGCGTGAGCTATGAGAAAGCGCCACGCTTCCCGAAGGGAGAA
AGGCGGACAGGTATCCGGTAAGCGGCAGGGTCGGAACAGGAGAGCGCACGAGGGAGCTTCCAGGGG
GAAACGCCTGGTATCTTTATAGTCCTGTCGGGTTTCGCCACCTCTGACTTGAGCGTCGATTTTTGT
GATGCTCGTCAGGGGGGCGGAGCCTATGGAAAAACGCCAGCAACGCGGCCTTTTTACGGTTCCTGG
CCTTTTGCTGGCCTTTTGCTCACATGTTCTTTCCTGCGTTATCCCCTGATTCTGTGGATAACCGTA
TTACCGCCTTTGAGTGAGCTGATACCGCTCGCCGCAGCCGAACGACCGAGCGCAGCGAGTCAGTGA
GCGAGGAAGCGGAAGAGCGCCCAATACGCAAACCGCCTCTCCCCGCGCGTTGGCCGATTCATTAAT
GCAGCTGGCACGACAGGTTTCCCGACTGGAAAGCGGGCAGTGAGCGCAACGCAATTAATGTGAGTT
AGCTCACTCATTAGGCACCCCAGGCTTTACACTTTATGCTTCCGGCTCGTATGTTGTGTGGAATTG
TGAGCGGATAACAATTTCACACAGGAAACAGCTATGACCATGATTACGCCAAGCGCGCAATTAACC
CTCACTAAAGGGAACAAAAGCTGGAGCTCCACCGCGGTGGCGGCCGCTCGAGTCTAGAGTTGTTTG
GATAATTAGATCTCTCTCTTTCTATCGTATTTTGCAATAATAGGTATTAACTTTTATACTGATTGT
TAGTAGATGCCTTCAAATTTTCTTTTTATTTAAATTCATATGCTATATCTTTTAAAACACTCCACA
TTTTATTGTTGCTAACTGTGCTATTGATCTTTAAGTCAATAGCTGCTCATTTTGTTGAACTCCACA
GAGACACTAAATTTGTTTATTTTGATGGATGCTTTATAATTAAAGTTACGTAATCTGCTTGACATT
TAGCCAACTATATAAAAAAGATCAAAATGTAGCTTAAATCTCAAAAAATCATCATAATTTACTATC
AAATTATTAAGAAATTCATATAATCACCACTTTATTGACTTTTATTCATCTTATAGAGTGATAGTA
GAGTTGAGCCAAATTGATACTTGTTTACGTTGTATTATTTTGAAATTTTAAAAAAATGAAAATGAG
AGAAAAATTTATTTAAATTTGAGCTTAGAATCTTTAAGGAAGATCAAAAATGGGCTAACTAAATGT
TAGAGTACGAAGACTGTTCTTGAAATAAAGTGTCATCCTTTACGAATCAAGTTGCTACTTTAATGA
ATAATAGAATTTGAGGTAGAGCTAAAATGAGAGATATAGTAATGCTATTGGATTATATTTGGTTTG
TATGATGGTTTTTCTTTGGTAAATGAATGATATAAATGAAGAGTGGCAATAAAATTAATTGAAATT

GAATGAAAAAAATGAATAGAAATTAAAGAAGAGTATAATTTTATTTTTGAATTTTATTTAAATTTTA
ATGCGTGTATTTATTTGGGTGATGTCGACTTGATATCTTCAAAGTATGGATTAATTATTTCAAATT
ATTAGAAGGTAATTAATCTGCATAAATTCAAAACTATAAAAATAAAACATTAAAATTAATTCAACC
TTATTGAAGCATCAAAATCTGAATCTCTAGAAAGACTGATTCTGATTGGATAATTTTTCGGCGCTA
AGGATTTTGGATTAAAGAAAATTAGATTTAATTATTAATCATGATTTGAATAGGATAGCAAGAATA
TTTGTTTGGTTTAAAAGGGAAAGCGGGTAATTATCAAAAATTTATAAATAATTTTAAAACAATAAA
TAGAAAAACAAATAAGATTATAAAAACTTACAAAAATGATTGAACAAGATGGTTTACACGCTGGTT
CTCCCGCCGCTTGGGTCGAAAGACTTTTCGGTTATGACTGGGCTCAACAAACCATCGGTTGCTCTG
ATGCCGCCGTCTTCCGTCTTTCTGCTCAAGGTCGTCCTGTTCTTTTCGTCAAGACCGACCTTTCTG
GTGCCCTTAATGAACTTCAAGATGAAGCTGCCCGTCTTTCTTGGCTTGCCACCACCGGTGTTCCTT
GCGCTGCTGTCCTTGACGTTGTCACTGAAGCCGGTAGAGACTGGCTTCTTTTAGGTGAAGTCCCCG
GTCGAGATCTTCTTTCTTCTCACCTTGCTCCTGCCGAAAAAGTTTCTATCATGGCTGATGCTATGC
GTCGTCTTCATACCCTTGATCCCGCTACCTGCCCTTTCGACCACCAAGCCAAACATCGTATCGAAC
GTGCTCGTACTCGTATGGAAGCCGGTCTTGTCGATCAAGATGATCTTGACGAAGAACATCAAGGTC
TTGCCCCTGCCGAACTTTTCGCCAGACTTAAGGCCCGTATGCCCGACGGTGAAGATCTTGTCGTCA
CCCATGGTGATGCCTGCTTACCCAATATCATGGTTGAAAATGGTCGTTTTTCTGGTTTCATCGACT
GTGGTCGTCTTGGTGTCGCCGACCGTTATCAAGATATTGCCTTAGCTACCCGTGATATCGCTGAAG
AACTTGGTGGTGAATGGGCTGACCGTTTCCTTGTCCTTTACGGTATCGCCGCTCCCGATTCTCAAC
GTATCGCCTTCTATCGTCTTCTTGACGAATTCTTCTGAGATCCTTAAATTAAAAATTCAATATATA
TTTACAAACTTTCATATAAAATAAATATATTATATAAAATTAATTTTTAGTGTATTATATTAACAT
TAAAGCACCAAAAAAACGTGTTAATATACTACTATAAAATATAATTTATTCCAAATTGACTAAAAT
CATTATTTTACAACTCATTTGTATATATATTTTATGTCAATTATTTTTTTTAACTTTCTAAAAAAA
AAAATTCCTCTTCACATACATGTTAGCTCTTAAAAATTTGTCTGCAAATCCAATAATAATATTTTT
TTTTGCCATTAAATTTTCAAATTTTTACTGGAAAAATGCAGCCCGGGGGATCAGACAATTTATTTC
TAAAAAATATTTAAAAATAAAAAATAATAAGGGTTTTGAATAACTCCTTTAATTTAAATACACATT
TTTAAATTTTTTTAGCTCTTTAAATATTCATAAAAATAAAAAATAACTAACTAAAAATAAATAAA
AAGATAATAATGATTAAAGGTATAATACTGAATAAGAAAAAACATAATAGAGTACTTATTTTTTAT
ATCACTATTTTTAATATCTTGAAAGCAAAACTTTTTTATATATCTTAAAATATATTGTATCGTTTA
TTCAATTATTTTCTTTAAATTTCAAATATATTGATAAAAAAGATGACATGTTTTTTAAAGAAAACA
TGAAATATAAAATAGATAAATATCAATTATTTTATTTATTAAATATATAAGCTGCTCAAAACATAG
CTCATTCATCAATTATAATATGTGAATCATTAATTTTCAAAATATTACTCATTATTTAGGCTATCA
TTTATTTTTTATTTTCAATTATCCGTTTCTATTATATTTTAATATTAAGTTGTGATTCTTGAATTT
TGTGTCATGAATTATTTGTAAATCTTTTTATTTCTGATAAAAAATATAAATTGATTGACTCATGAT
TTAAATCATGAGTCAACCTAACTAATTTTCAAAATTCTTCTATTCTAAAATATAGATGTGATTCTT
GAATCTCTCTTGAATATAAAGTAATTTTTTATATTTCTGATATAATTCTTAGCTACGTGATTCACG
ATTTATGCAATGATCCATATAAAATAATGTAAATAGTGTATATATATATATTCGTCTTTTTTATTC

TTTATATAATTTAAAAAAATTAAAAAAATTTAATAAAGCTCTAATAAAATAAATAATAATACTAAA
CTTAAACATCCCGGGGGATCCAAATTGTTCTACAAACCGGGCGCCTGCTCTCTCGCTTCCCATATC
ACCCTGCGTGAGAGCGGAAAGGATTTTACGCTCGTCAGTGTGGATTTAATGAAAAACGTCTCGAA
AACGGTGACGATTACTTTGCCGTTAACCCTAAGGGACAGGTGCCTGCATTGCTGCTGGACGATGGT
ACTTTGCTGACGGAAGGCGTAGCAATTATGCAGTATCTTGCCGACAGCGTCCCCGACCGCCAGTTG
CTGGCACCGGTAAACAGTATTTCCCGCTATAAAACCATCGAATGGCTGAATTACATCGCCACCGAG
CTGCATAAAGGCTTCACACCTCTGTTTCGCCCTGATACACCGGAAGAGTACAAACCAACAGTTCGC
GCGCAGCTGGAGAAGAAACTGCAATATGTGAACGATGCTCTGAAGGATGAGCACTGGATCTGCGGG
CAACGATTTACAATTGCTGATGCCTATCTGTTTACGGTTCTGCGCTGGGCATACGCGGTGAAACTG
AATCTGGAAGGGTTAGAGTACATTGCGGCATTTATGCAACATATGGCTGAACGTCCGGAAGTACAA
GACGCGCTGTCAGCGGAAGGCTTAAAG**GGATCCGTAAGTAACGGATATGCAAAAGtaaacaaattt**
taggcttttataaaaaataggaagcaggccggcggTAGATAAAAGAAGAATTCAAAGGCTGCAGct
cgtcagggggcggagcctatggaaaaacgccagcaacgcggcctttttacggttcctggcctttt
gctggccttttgctcacatgttctttcctgcgttatcccctgattctgtggataaccgtattaccg
cctttgagtgagctgataccgctcgccgcagccgaacgaccgagcgcagcgagtcagtgagcgagg
aagcggaagagcgcccaatacgcaaaccgcctctccccgcgcgttggccgattcattaatgcagct
ggcacgacaggtttcccgactggaaagcgggcagtgagcgcaacgcaattaatgtgagttagctca
ctcattaggcaccccaggctttacactttatgcttccggctcgtatgttgtgtggaattgtgagcg
gataacaatttcacacaggaaacagctCTGCAGGCGAACTGAATCGGTCAGCTAAACCAACCAATC
AACATAATAAACTTTATTATTTTTACTTAAGCATCTTACTGTTGTTGTAATAGTAGAGAAAGAAAT
ACCCAATTAACTTCATTCACATAACATTAATATCTATAAACATCTTTTTTCTCACATATATACAAC
TCTCTAAATCAACAAATAACTTTTTAAAAATAATGGATATATATTAACAAAATAATATATCTCTTT
TTACAAAATAGTTCTTATATAAATACGTATTCTGCACTCACCCGCATTTTTCACAACAAAAACATA
CCAAAAAAATTCTTACTTCTACATGTTTCCTTTCTTATTATTACAAAATTATTTTATAAATAGCAT
ACAAAAATAAATACAATAAAAAAATAAACAAAATCCTTTTTTATTTTGAATTATTTAAAACAAATA
TTTTCAATCAATCAGTCAGTCAGCATAATATTAAAGCAACAAAACAAACCCAAGTTGTTTTTATAG
TTTTTTAATTGCTTTTCAGTACTATAAATAAATTTGTTATTACTTCAAGATTGATAAACTTCTTTT
TTAAATTAAATATCTATGAATGAATAAATAAGTTGATATCTCTTTTTAACTTGTTTTCCTCTCTTT
TACTTACTTGCCAATTTTTTTTTAAATTAAAGAAATATCTTTTTATTTTTCAAAAACAAAATTTA
TTTTCCCTTGTATACAAAAACCCCTTTATTTAAATAAAATCTTTATGCCCATCAATAGCCACATCT
TCTCGAGGGGGGGCCCGGTACCCAATTCGCCCTATAGTGAGTCGTATTACGCGCGCTCACTGGCCG
TCGTTTTACAACGTCGTGACTGGGAAAACCCTGGCGTTACCCAACTTAATCGCCTTGCAGCACATC
CCCCTTTCGCCAGCTGGCGTAATAGCGAAGAGGCCCGCACCGATCGCCCTTCCCAACAGTTGCGCA
GCCTGAATGGCGAATGGGACGCGCCCTGTAGCGGCGCATTAAGCGCGGCGGGTGTGGTGGTTACGC
GCAGCGTGACCGCTACACTTGCCAGCGCCCTAGCGCCCGCTCCTTTCGCTTTCTTCCCTTCCTTTC
TCGCCACGTTCGCCGGCTTTCCCCGTCAAGCTCTAAATCGGGGGCTCCCTTTAGGGTTCCGATTTA

GTGCTTTACGGCACCTCGACCCCAAAAAACTTGATTAGGGTGATGGTTCACGTAGTGGGCCATCGC
CCTGATAGACGGTTTTTCGCCCTTTGACGTTGGAGTCCACGTTCTTTAATAGTGGACTCTTGTTCC
AAACTGGAACAACACTCAACCCTATCTCGGTCTATTCTTTTGATTTATAAGGGATTTTGCCGATTT
CGGCCTATTGGTTAAAAAATGAGCTGATTTAACAAAAATTTAACGCGAATTTTAACAAAATATTAA
CGCTTACAATTTAG
