# Supplementary material for: Resistance to 6-Methylpurine is Conferred by Defective Adenine Phosphoribosyltransferase in Tetrahymena
Source: Genes (Basel). 2018 Mar 23;9(4):179. doi: 10.3390/genes9040179 (PMC5924521; doi:10.3390/genes9040179)
Supplement: Supplementary file 1 [file genes-09-00179-s001.zip › Figure S5.pdf]

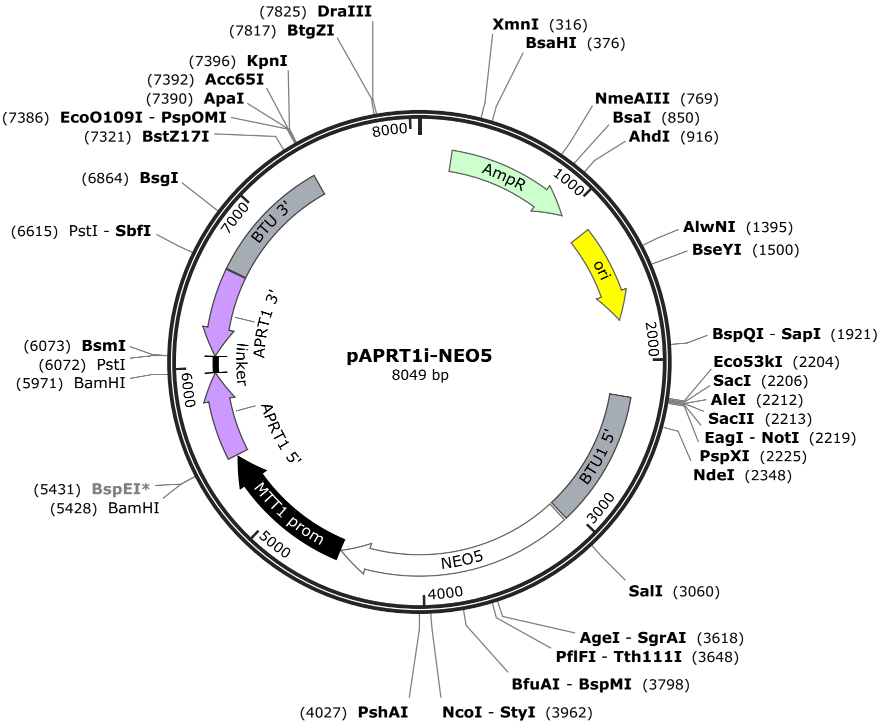

Figure S5. Map of the *APRT1* knockdown plasmid pAPRT1i-NEO5, with paromomycin resistance cassette (*NEO5*), cadmium-inducible *MTT1* promoter, hairpin RNAi cassette, and 5′ and 3′ UTRs of the MAC *BTU1*.

5′ and 3′ UTRs of BTU1

ACGT: APRT1 CDS

NEO5 cassette

MTT1 promoter

Linker

GTGGCACTTTTCGGGGAAATGTGCGCGGAACCCCTATTTGTTTATTTTTCTAAATACATTCAAATA
TGTATCCGCTCATGAGACAATAACCCTGATAAATGCTTCAATAATATTGAAAAAGGAAGAGTATGA
GTATTCAACATTTCCGTGTCGCCCTTATTCCCTTTTTTGCGGCATTTTGCCTTCCTGTTTTTGCTC
ACCCAGAAACGCTGGTGAAAGTAAAAGATGCTGAAGATCAGTTGGGTGCACGAGTGGGTTACATCG
AACTGGATCTCAACAGCGGTAAGATCCTTGAGAGTTTTCGCCCCGAAGAACGTTTTCCAATGATGA
GCACTTTTAAAGTTCTGCTATGTGGCGCGGTATTATCCCGTATTGACGCCGGGCAAGAGCAACTCG
GTCGCCGCATACACTATTCTCAGAATGACTTGGTTGAGTACTCACCAGTCACAGAAAAGCATCTTA
CGGATGGCATGACAGTAAGAGAATTATGCAGTGCTGCCATAACCATGAGTGATAACACTGCGGCCA
ACTTACTTCTGACAACGATCGGAGGACCGAAGGAGCTAACCGCTTTTTTGCACAACATGGGGGATC
ATGTAACTCGCCTTGATCGTTGGGAACCGGAGCTGAATGAAGCCATACCAAACGACGAGCGTGACA
CCACGATGCCTGTAGCAATGGCAACAACGTTGCGCAAACTATTAACTGGCGAACTACTTACTCTAG

CTTCCCGGCAACAATTAATAGACTGGATGGAGGCGGATAAAGTTGCAGGACCACTTCTGCGCTCGG
CCCTTCCGGCTGGCTGGTTTATTGCTGATAAATCTGGAGCCGGTGAGCGTGGGTCTCGCGGTATCA
TTGCAGCACTGGGGCCAGATGGTAAGCCCTCCCGTATCGTAGTTATCTACACGACGGGGAGTCAGG
CAACTATGGATGAACGAAATAGACAGATCGCTGAGATAGGTGCCTCACTGATTAAGCATTGGTAAC
TGTCAGACCAAGTTTACTCATATATACTTTAGATTGATTTAAAACTTCATTTTTAATTTAAAAGGA
TCTAGGTGAAGATCCTTTTTGATAATCTCATGACCAAAATCCCTTAACGTGAGTTTTCGTTCCACT
GAGCGTCAGACCCCGTAGAAAAGATCAAAGGATCTTCTTGAGATCCTTTTTTTCTGCGCGTAATCT
GCTGCTTGCAAACAAAAAAACCACCGCTACCAGCGGTGGTTTGTTTGCCGGATCAAGAGCTACCAA
CTCTTTTTCCGAAGGTAACTGGCTTCAGCAGAGCGCAGATACCAAATACTGTCCTTCTAGTGTAGC
CGTAGTTAGGCCACCACTTCAAGAACTCTGTAGCACCGCCTACATACCTCGCTCTGCTAATCCTGT
TACCAGTGGCTGCTGCCAGTGGCGATAAGTCGTGTCTTACCGGGTTGGACTCAAGACGATAGTTAC
CGGATAAGGCGCAGCGGTCGGGCTGAACGGGGGGTTCGTGCACACAGCCCAGCTTGGAGCGAACGA
CCTACACCGAACTGAGATACCTACAGCGTGAGCTATGAGAAAGCGCCACGCTTCCCGAAGGGAGAA
AGGCGGACAGGTATCCGGTAAGCGGCAGGGTCGGAACAGGAGAGCGCACGAGGGAGCTTCCAGGGG
GAAACGCCTGGTATCTTTATAGTCCTGTCGGGTTTCGCCACCTCTGACTTGAGCGTCGATTTTTGT
GATGCTCGTCAGGGGGGCGGAGCCTATGGAAAAACGCCAGCAACGCGGCCTTTTTACGGTTCCTGG
CCTTTTGCTGGCCTTTTGCTCACATGTTCTTTCCTGCGTTATCCCCTGATTCTGTGGATAACCGTA
TTACCGCCTTTGAGTGAGCTGATACCGCTCGCCGCAGCCGAACGACCGAGCGCAGCGAGTCAGTGA
GCGAGGAAGCGGAAGAGCGCCCAATACGCAAACCGCCTCTCCCCGCGCGTTGGCCGATTCATTAAT
GCAGCTGGCACGACAGGTTTCCCGACTGGAAAGCGGGCAGTGAGCGCAACGCAATTAATGTGAGTT
AGCTCACTCATTAGGCACCCCAGGCTTTACACTTTATGCTTCCGGCTCGTATGTTGTGTGGAATTG
TGAGCGGATAACAATTTCACACAGGAAACAGCTATGACCATGATTACGCCAAGCGCGCAATTAACC
CTCACTAAAGGGAACAAAAGCTGGAGCTCCACCGCGGTGGCGGCCGCTCGAGTCTAGAGTTGTTTG
GATAATTAGATCTCTCTCTTTCTATCGTATTTTGCAATAATAGGTATTAACTTTTATACTGATTGT
TAGTAGATGCCTTCAAATTTTCTTTTTATTTAAATTCATATGCTATATCTTTTAAAACACTCCACA
TTTTATTGTTGCTAACTGTGCTATTGATCTTTAAGTCAATAGCTGCTCATTTTGTTGAACTCCACA
GAGACACTAAATTTGTTTATTTTGATGGATGCTTTATAATTAAAGTTACGTAATCTGCTTGACATT
TAGCCAACTATATAAAAAAGATCAAAATGTAGCTTAAATCTCAAAAAATCATCATAATTTACTATC
AAATTATTAAGAAATTCATATAATCACCACTTTATTGACTTTTATTCATCTTATAGAGTGATAGTA
GAGTTGAGCCAAATTGATACTTGTTTACGTTGTATTATTTTGAAATTTTAAAAAAATGAAAATGAG
AGAAAAATTTATTTAAATTTGAGCTTAGAATCTTTAAGGAAGATCAAAAATGGGCTAACTAAATGT
TAGAGTACGAAGACTGTTCTTGAAATAAAGTGTCATCCTTTACGAATCAAGTTGCTACTTTAATGA
ATAATAGAATTTGAGGTAGAGCTAAAATGAGAGATATAGTAATGCTATTGGATTATATTTGGTTTG
TATGATGGTTTTTCTTTGGTAAATGAATGATATAAATGAAGAGTGGCAATAAAATTAATTGAAATT
GAATGAAAAAATGAATAGAAATTAAAGAAGAGTATAATTTTATTTTTGAATTTTATTTAAATTTTA
ATGCGTGTATTTATTTGGGTGATGTCGACTTGATATCTTCAAAGTATGGATTAATTATTTCAAATT
ATTAGAAGGTAATTAATCTGCATAAATTCAAAACTATAAAAATAAAACATTAAAATTAATTCAACC
TTATTGAAGCATCAAAATCTGAATCTCTAGAAAGACTGATTCTGATTGGATAATTTTTCGGCGCTA
AGGATTTTGGATTAAAGAAAATTAGATTTAATTATTAATCATGATTTGAATAGGATAGCAAGAATA
TTTGTTTGGTTTAAAAGGGAAAGCGGGTAATTATCAAAAATTTATAAATAATTTTAAAACAATAAA
TAGAAAAACAAATAAGATTATAAAAACTTACAAAAATGATTGAACAAGATGGTTTACACGCTGGTT
CTCCCGCCGCTTGGGTCGAAAGACTTTTCGGTTATGACTGGGCTCAACAAACCATCGGTTGCTCTG
ATGCCGCCGTCTTCCGTCTTTCTGCTCAAGGTCGTCCTGTTCTTTTCGTCAAGACCGACCTTTCTG
GTGCCCTTAATGAACTTCAAGATGAAGCTGCCCGTCTTTCTTGGCTTGCCACCACCGGTGTTCCTT

GCGCTGCTGTCCTTGACGTTGTCACTGAAGCCGGTAGAGACTGGCTTCTTTTAGGTGAAGTCCCCG
GTCGAGATCTTCTTTCTTCTCACCTTGCTCCTGCCGAAAAAGTTTCTATCATGGCTGATGCTATGC
GTCGTCTTCATACCCTTGATCCCGCTACCTGCCCTTTCGACCACCAAGCCAAACATCGTATCGAAC
GTGCTCGTACTCGTATGGAAGCCGGTCTTGTCGATCAAGATGATCTTGACGAAGAACATCAAGGTC
TTGCCCCTGCCGAACTTTTCGCCAGACTTAAGGCCCGTATGCCCGACGGTGAAGATCTTGTCGTCA
CCCATGGTGATGCCTGCTTACCCAATATCATGGTTGAAAATGGTCGTTTTTCTGGTTTCATCGACT
GTGGTCGTCTTGGTGTCGCCGACCGTTATCAAGATATTGCCTTAGCTACCCGTGATATCGCTGAAG
AACTTGGTGGTGAATGGGCTGACCGTTTCCTTGTCCTTTACGGTATCGCCGCTCCCGATTCTCAAC
GTATCGCCTTCTATCGTCTTCTTGACGAATTCTTCTGAGATCCTTAAATTAAAAATTCAATATATA
TTTACAAACTTTCATATAAAATAAATATATTATATAAAATTAATTTTAGTGTATTATATTAACAT
TAAAGCACCAAAAAAACGTGTTAATATACTACTATAAAATATAATTTATTCCAAATTGACTAAAAT
CATTATTTTACAACTCATTTGTATATATATTTTATGTCAATTATTTTTTTTAACTTTCTAAAAAAA
AAAATTCCTCTTCACATACATGTTAGCTCTTAAAAATTTGTCTGCAAATCCAATAATAATATTTTT
TTTTGCCATTAAATTTTCAAATTTTTACTGGAAAAATGCAGCCCGGGGGATCAGACAATTTATTTC
TAAAAAATATTTAAAAATAAAAAATAATAAGGGTTTTGAATAACTCCTTTAATTTAAATACACATT
TTTAAATTTTTTTAGCTCTTTAAATATTCATAAAAATAAAAAATAACTAACTAAAAATAAATAAA
AAGATAATAATGATTAAAGGTATAATACTGAATAAGAAAAAACATAATAGAGTACTTATTTTTTAT
ATCACTATTTTTAATATCTTGAAAGCAAAACTTTTTTATATATCTTAAAATATATTGTATCGTTTA
TTCAATTATTTTCTTTAAATTTCAAATATATTGATAAAAAAGATGACATGTTTTTTAAAGAAAACA
TGAAATATAAAATAGATAAATATCAATTATTTTATTTATTAAATATATAAGCTGCTCAAAACATAG
CTCATTCATCAATTATAATATGTGAATCATTAATTTTCAAAATATTACTCATTATTTAGGCTATCA
TTTATTTTTTATTTTCAATTATCCGTTTCTATTATATTTTAATATTAAGTTGTGATTCTTGAATTT
TGTGTCATGAATTATTTGTAAATCTTTTTATTTCTGATAAAAAATATAAATTGATTGACTCATGAT
TTAAATCATGAGTCAACCTAACTAATTTTCAAAATTCTTCTATTCTAAAATATAGATGTGATTCTT
GAATCTCTCTTGAATATAAAGTAATTTTTTATATTTCTGATATAATTCTTAGCTACGTGATTCACG
ATTTATGCAATGATCCATATAAAATAATGTAAATAGTGTATATATATATATTCGTCTTTTTTATTC
TTTATATAATTTAAAAAAATTAAAAAAATTTAATAAAGCTCTAATAAAATAAATAATAATACTAAA
CTTAAACATCCCGGGGGATCCGGATTACTAAAATTAAGAAATAGGTAAAGTTATTAGCGAAAACTT
CAGAAATATTCCTGATTTCCCTTAACCTGGTATTATCTTTAAAGATATTGCTCCTCTCCTCTCTAA
TCCTGCCATTTTTAAGCGTATTACTGATGCTTTTGTAATTGCTTTGAAGGACATTGATTTTGATAA
AATTGTTATCTTAGAATCTAGAGGTTTTATTTTTGGAACCGCTATTGGTTTACAAATGGGAAAGGG
ATTAATTTTAATCAGAAAGAAAGGCAAACTACCTGGTAAGGTTGAATAATTCTCATTCGACTTAGA
ATACGGTAAGGATGTTTTTGAAATTCAAAGTGATGCTTTAGCTAAAGGTGAAAAAGTTGTTATTGT
TGATGATATTCTAGCTACTGGTGGAACTGCTGATGCAGCTTGCAGATTAGTTGAAAAGGTAGGAGG
AGTAGTATAAGCTGTTATGTTCCTTTACCAAATTAAGGATCTTAAGGGCGAAGAAAGATTATCTAA
ATACAAGGTTATTAATCTTCATATTGAATG**GGATCCGTAAGTAACGGATATGCAAAAGtaaacaaa**
ttttaggcttttataaaaaataggaagcaggccggcggTAGATAAAAGAAGAATTCAAAGGCTGCA
GCATTCAATATGAAGATTAATAACCTTGTATTTAGATAATCTTTCTTCGCCCTTAAGATCCTTAAT
TTGGTAAAGGAACATAACAGCTTATACTACTCCTCCTACCTTTTCAACTAATCTGCAAGCTGCATC
AGCAGTTCCACCAGTAGCTAGAATATCATCAACAATAACAACTTTTTCACCTTTAGCTAAAGCATC
ACTTTGAATTTCAAAAACATCCTTACCGTATTCTAAGTCGAATGAGAATTATTCAACCTTACCAGG
TAGTTTGCCTTTCTTTCTGATTAAAATTAATCCCTTTCCCATTTGTAAACCAATAGCGGTTCCAAA
AATAAAACCTCTAGATTCTAAGATAACAATTTTATCAAAATCAATGTCCTTCAAAGCAATTACAAA
AGCATCAGTAATACGCTTAAAAATGGCAGGATTAGAGAGGAGAGGAGCAATATCTTTAAAGATAAT

ACCAGGTTAAGGGAAATCAGGAATATTTCTGAAGTTTTCGCTAATAACTTTACCTATTTCTTAATT
TTAGTAATCCCTGCAGGCGAACTGAATCGGTCAGCTAAACCAACCAATCAACATAATAAACTTTAT
TATTTTTACTTAAGCATCTTACTGTTGTTGTAATAGTAGAGAAAGAAATACCCAATTAACTTCATT
CACATAACATTAATATCTATAAACATCTTTTTTCTCACATATATACAACTCTCTAAATCAACAAAT
AACTTTTTAAAAATAATGGATATATATTAACAAAATAATATATCTCTTTTTACAAAATAGTTCTTA
TATAAATACGTATTCTGCACTCACCCGCATTTTTCACAACAAAAACATACCAAAAAAATTCTTACT
TCTACATGTTTCCTTTCTTATTATTACAAAATTATTTTATAAATAGCATACAAAAATAAATACAAT
AAAAAAATAAACAAAATCCTTTTTTATTTTGAATTATTTAAAACAAATATTTTCAATCAATCAGTC
AGTCAGCATAATATTAAAGCAACAAAACAAACCCAAGTTGTTTTTATAGTTTTTTAATTGCTTTTC
AGTACTATAAATAAATTTGTTATTACTTCAAGATTGATAAACTTCTTTTTTAAATTAAATATCTAT
GAATGAATAAATAAGTTGATATCTCTTTTTAACTTGTTTTCCTCTCTTTTACTTACTTGCCAATTT
TTTTTTTAAATTAAAGAAATATCTTTTTATTTTTCAAAAACAAAATTTATTTTCCCTTGTATACAA
AAACCCCTTTATTTAAATAAAATCTTTATGCCCATCAATAGCCACATCTTCTCGAGGGGGGCCCG
GTACCCAATTCGCCCTATAGTGAGTCGTATTACGCGCGCTCACTGGCCGTCGTTTTACAACGTCGT
GACTGGGAAAACCCTGGCGTTACCCAACTTAATCGCCTTGCAGCACATCCCCCTTTCGCCAGCTGG
CGTAATAGCGAAGAGGCCCGCACCGATCGCCCTTCCCAACAGTTGCGCAGCCTGAATGGCGAATGG
GACGCGCCCTGTAGCGGCGCATTAAGCGCGGCGGGTGTGGTGGTTACGCGCAGCGTGACCGCTACA
CTTGCCAGCGCCCTAGCGCCCGCTCCTTTCGCTTTCTTCCCTTCCTTTCTCGCCACGTTCGCCGGC
TTTCCCCGTCAAGCTCTAAATCGGGGGCTCCCTTTAGGGTTCCGATTTAGTGCTTTACGGCACCTC
GACCCCAAAAAACTTGATTAGGGTGATGGTTCACGTAGTGGGCCATCGCCCTGATAGACGGTTTTT
CGCCCTTTGACGTTGGAGTCCACGTTCTTTAATAGTGGACTCTTGTTCCAAACTGGAACAACACTC
AACCCTATCTCGGTCTATTCTTTTGATTTATAAGGGATTTTGCCGATTTCGGCCTATTGGTTAAAA
AATGAGCTGATTTAACAAAAATTTAACGCGAATTTTAACAAAATATTAACGCTTACAATTTAG
